# Characterizing circulating biomarkers for childhood dementia disorders: A scoping review of clinical trials

**DOI:** 10.1016/j.neurot.2025.e00546

**Published:** 2025-02-12

**Authors:** Arlene D'Silva, James Barnes, Jason Djafar, Kaustuv Bhattacharya, Jingya Yan, Shekeeb Mohammad, Sushil Bandodkar, Alexandra Johnson, Michel Tchan, Christina Miteff, Kristina L. Elvidge, Russell C. Dale, Michelle Farrar

**Affiliations:** a Department of Neurology, The Sydney Children's Hospitals Network, Sydney, Australia; b Discipline of Paediatrics and Child Health, School of Clinical Medicine, UNSW Medicine and Health, The University of New South Wales, Sydney, Australia; c UNSW RNA Institute, The University of New South Wales, Sydney, Australia; d Sydney Children's Hospitals' Network, Westmead, NSW 2145, Australia; e Clinical School, The Children's Hospital at Westmead, Faculty of Medicine and Health, University of Sydney, NSW, Australia; f Kids Neuroscience Centre, The Children's Hospital at Westmead, Faculty of Medicine and Health, University of Sydney, Clinical School, NSW, Australia; g Department of Genetic Medicine, Westmead Hospital, Westmead, NSW 2145, Australia; h Faculty of Medicine and Health, University of Sydney, NSW, Australia; i Children, Young People and Families Directorate of Hunter New England Local Health District and John Hunter Children's Hospital, New Lambton Heights, NSW 2305, Australia; j Childhood Dementia Initiative, Brookvale, NSW, 2100, Australia

**Keywords:** Biomarkers, Childhood dementia, Clinical trials, Endpoints, Metabolomics, Proteomics

## Abstract

Childhood dementias, a group of neurological disorders are characterised by neurocognitive decline, with physical and psychosocial impacts for individuals. With therapy available for <5 ​% of childhood dementias, there is a high level of unmet need. Integration of biomarkers in clinical trials are important to characterize distinctive biological activities and interrogate targets for therapeutic development. This study reviewed four clinical trial registries to examine circulating biomarkers in childhood dementias. Findings from 262 studies were synthesized across 49/72 (68 ​%) childhood dementia disorders. Disease-related biomarkers were associated with 1) the primary pathophysiology 2) downstream pathogenic events 3) drug-related pharmacokinetics, safety and/or tolerability. The predominant biological measures were metabolites linked to the primary pathophysiological pathway (102 measures, 185 studies), while use of cytoskeletal proteins (3 measures, 15 studies), inflammatory mediators (19 measures, 24 studies), oxidative stress-related analytes (15 measures, 8 studies), neurotransmitters or related neuro-metabolites (3 measures, 5 studies) were limited. A range of potential biomarkers are used in clinical trials; however, their use is inconsistent and under utilised among conditions. Development of a panel of biomarkers has potential to interrogate and link shared biological pathways across the heterogeneity of childhood dementias to exert a significant impact for the development of disease-modifying therapies.

## Introduction

Childhood dementia is a heterogeneous group of neurological disorders defined by progressive neurocognitive decline, and a constellation of physical, psychological, social, and economic impacts for children, adolescents, and their families [1]. Aligning with adult dementia, childhood dementia is not a single entity. Instead, it defines the sequalae of progressive brain damage caused by more than 140 rare and ultra rare genetic conditions, including inborn errors of metabolism, such as lysosomal, mitochondrial, and peroxisomal diseases, leukodystrophies and neurodegeneration with brain iron accumulation [2,3]. Childhood dementias have an estimated incidence of 1 in 2900 births and a median life expectancy of 9 years. With <5 ​% having approved disease modifying therapies, there is a paucity of targeted treatments for these progressive conditions [2].

The considerable heterogeneity in phenotype, disease progression and limited knowledge of natural history in childhood dementia conditions, coupled with small individual patient populations pose a significant challenge for therapeutic development. Selecting the appropriate clinical manifestations that may have greater or earlier responsiveness to treatment is critical [4], yet a broad range of neurodevelopmental ages, disease stages, and comorbidities hinder using unified practices to capture clinical outcomes, or endpoints. To address this gap, biomarker discovery is imperative, to characterize shared pathogenic pathways across the diverse range of conditions and to evaluate the effect of therapeutic intervention on common downstream manifestations of disease.

With the growth of advanced therapeutics including stem-cell, enzyme replacement, neuroimmunomodulation, and genetic therapies, the therapeutic development pipeline for childhood dementia is rapidly expanding and optimal study design is therefore required. Innovations in clinical trial design increase trial efficiencies and knowledge gain across rare diseases, with master protocols used to study multiple therapies or diseases, in contrast with single trials conducted independently [5]. These share key design components, such as parallel investigation of biomarker-matched therapies or cohorts and highlight the value of biomarkers to provide diagnostic, predictive, prognostic and pharmacodynamic data.

Conventional clinical trials are incorporating biomarkers as a surrogate endpoint of clinical benefit, highlighted by the recent acceleration of orphan drug approval of tofersen for *SOD-1* amyotrophic lateral sclerosis, founded on a biomarker based primary endpoint [[6], [7], [8], [9]]. Clinical trials may also benefit from biomarkers as secondary or exploratory endpoints, to substantiate proof of concept by demonstrating a biological response to a therapy, or to stratify patients with a greater potential to respond, especially when there may be clinical and genotypic cohort heterogeneity at baseline. For example, in adult dementias, phosphorylated tau protein has been used to differentiate between phenotypes in Alzheimer's disease, augmenting classification of disease stage, for the potential to target enrolment into clinical trials. Similarly, in childhood dementias, circulating biomarkers can facilitate assessment of diverse processes including immune function, metabolism, nucleic acids, and proteins with multi-omic technologies. These surrogates of disease activity and therapeutic response are especially attractive for use in a paediatric population as they are easier to access compared with invasive biopsies or neuroimaging procedures and may be more sensitive to detect early changes. The purpose of this scoping review was to assess the utility of circulating biomarkers in childhood dementia clinical trials. Delineating a set of putative biomarkers in childhood dementias is considered a foundation to develop and evaluate targeted treatments and provide access and opportunity to children who would most benefit from them.

## Methods

### Data sources and search strategy

This search was conducted through four electronic database registries of clinical trials: ClinicalTrials.gov (run by the US National Library of Medicine at the National Institutes of Health (NIH), established 29 Feb 2000), the Australia and New Zealand Clinical Trials Registry (anzctr.org.au, established 2005), the European Union Clinical Trials Register (clinicaltrialsregister.eu, established 1 May 2004) and the International Standard Randomised Controlled Trial Number Registry (established 2000).

A systematic search was performed of all study records registered from the establishment of each database up to 10 May 2023 using the Preferred Reporting Items for Systematic Review and Meta-Analysis extension for Scoping Reviews (PRISMA-ScR) framework [10]. To identify relevant studies two authors undertook independent searches. Search terms included individual disease names and alternative names for each childhood dementia disorder, obtained from the Childhood Dementia Knowledge base (https://www.childhooddementia.org/knowledgebase which included 72 childhood dementia conditions, accessed 01/May/2023, Appendix 1) [2,4]. (Medline, Scopus, and PubMed searches were undertaken using unique clinical trial identifier numbers of the clinical trials that met inclusion criteria to identify relevant publications to 19 Dec 2023. A study was considered completed at the time of censoring if its record neither specifically identified it as ongoing nor had a nominated end date after 31^st^ August 2023.

### Study selection

The resulting list of clinical trial titles and objectives from search results were examined and duplicates were consolidated and removed. Additional study details were obtained and assessed regarding eligibility criteria. Observational and interventional clinical studies of humans were included if they incorporated participants less than 18 years of age with a childhood dementia disorder, were reported in English, and included circulating biological markers (blood, urine, or cerebrospinal fluid) as either inclusion criteria, endpoints, primary outcome measures, secondary outcome measures, or exploratory measures. All clinical trial study designs and phases were included. Clinical trials that mentioned biomarker repository without explicitly stating the biomarker, studies only focusing on adult populations and studies that exclusively investigated non-CNS systemic measures, without any neurological interrogations, or only non-circulatory biomarkers were excluded.

### Data extraction

For studies meeting the eligibility criteria, data were extracted into an excel spreadsheet and the following were collated: population (disease names, ages of enrolment), intervention (study type and design, estimated sample size, investigational product, study status), outcome measures (primary, secondary, and exploratory, endpoints, details of biological measures) and reporting of study results. All studies and extracted data were verified by a second researcher for accuracy and completeness. Data extraction differences were resolved by discussion until a consensus was reached.

### Data analysis and synthesis

Data analysis comprised several phases: (1) three authors independently reviewed and summarized biomarker applications in clinical studies, noting common approaches and salient themes; (2) researchers collectively generated a conceptual framework and a set of agreed themes; (3) coding of biomarkers in clinical studies from these themes was conducted; (4) in a second cycle of coding, subthemes were generated and a narrative synthesis was completed according to the aims of this scoping review. For completed studies in which details of biomarker use were publicised at the time of censoring, the role of each biomarker was classified by the authors in accordance with the Biomarkers, EndpointS, and other Tools (BEST) resource glossary [11]. The authors met regularly throughout the data collection, coding, and analysis phases to ensure reflexivity. Coding and analysis were led by two researchers (AD and JD) with coding reviewed by (JB and MF).

## Results

**Fig. 1:**
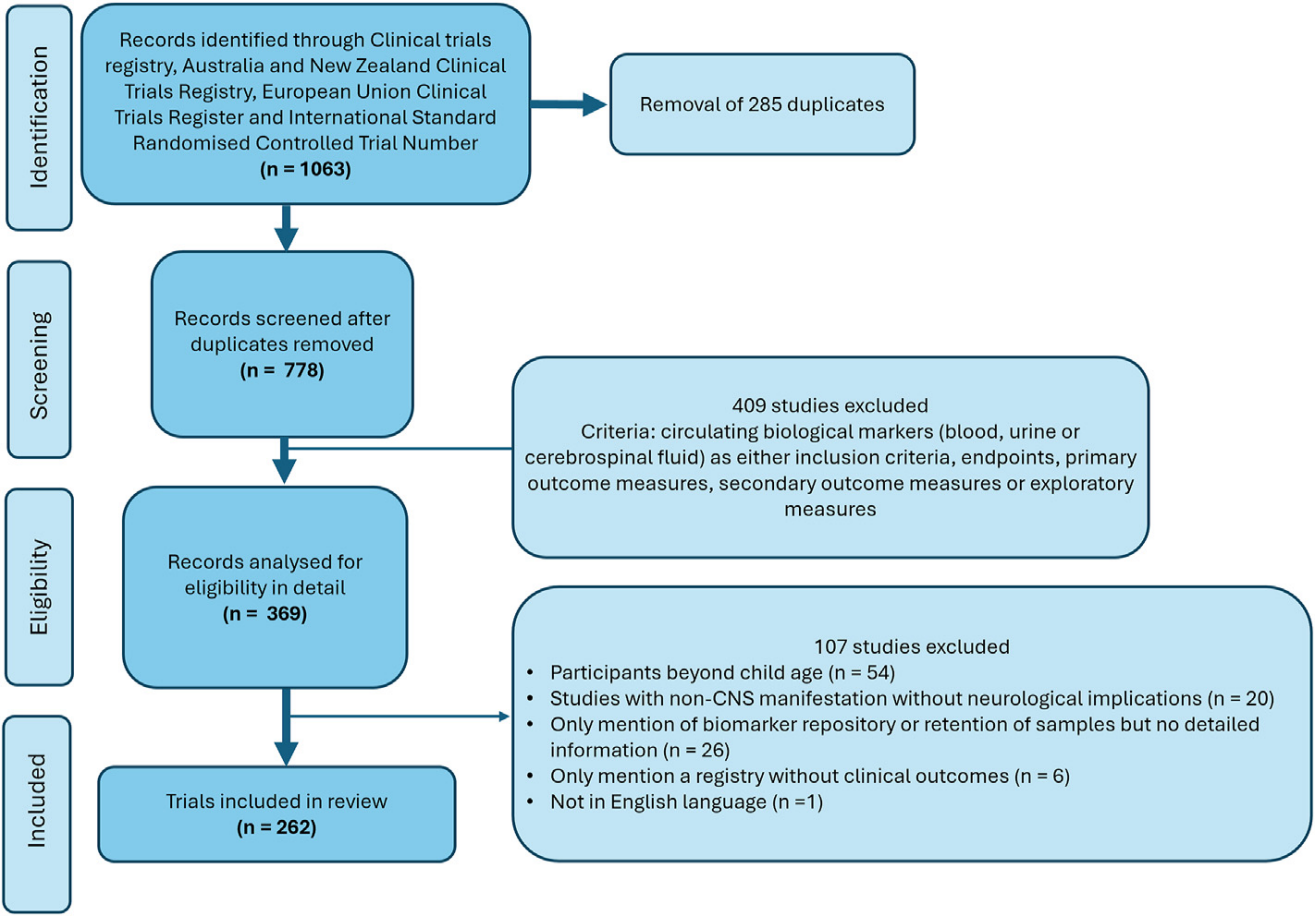
PRISMA flow diagram - exclusion and inclusion criteria for studies reviewed.

Initial database searching yielded 1063 clinical studies registered, of which 285 were duplicates. Of the 778 unique clinical studies, 262 met all inclusion criteria (Fig. 1).

### Study characteristics

**Fig. 2:**
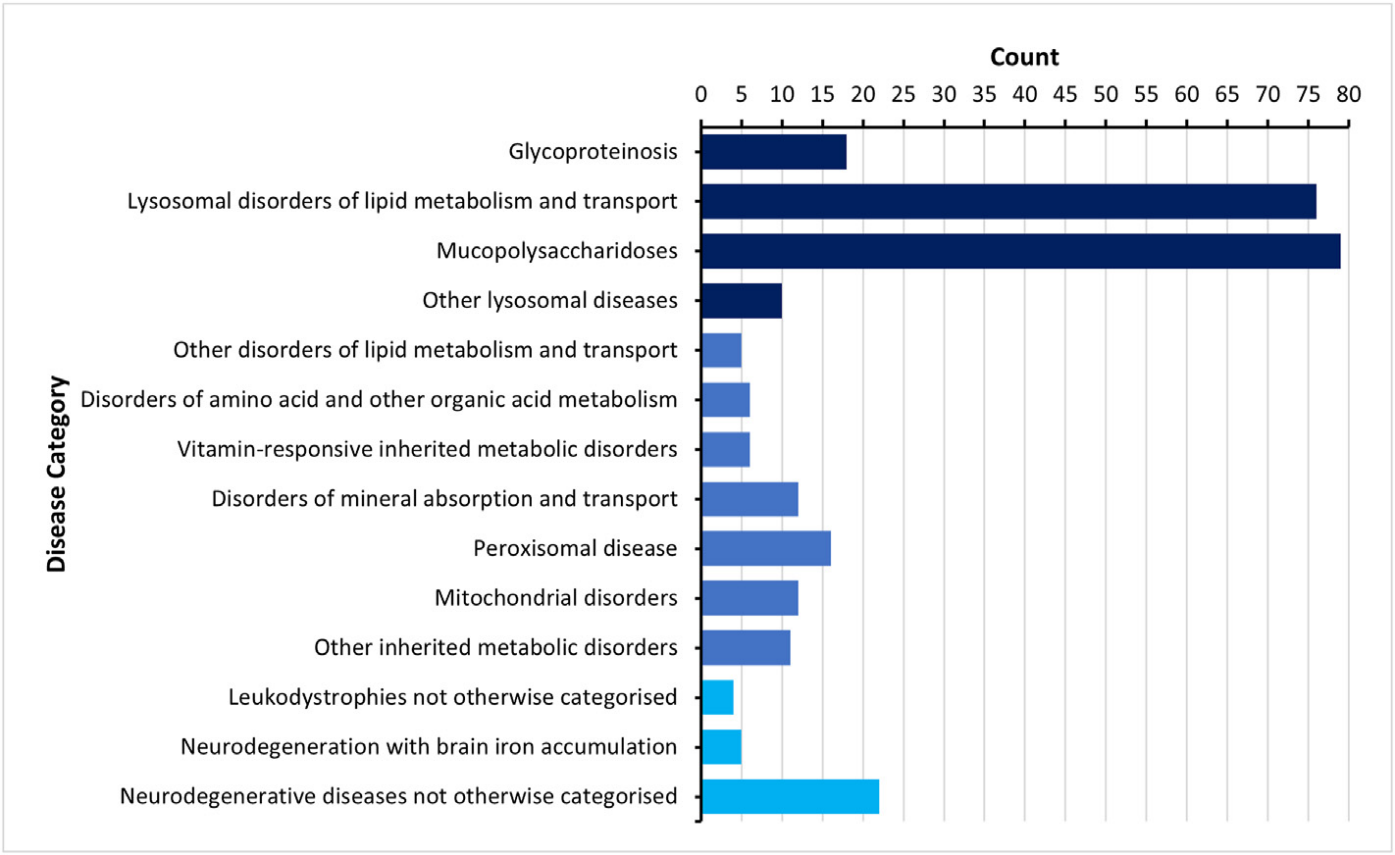
**The number of clinical trials incorporating circulating biomarkers in childhood dementia disorders.** The individual conditions are listed, according to their disease categories (**Inborn Errors of Metabolism**, their subset **Lysosomal Storage Disorders**, and those **Otherwise Classified**), as follows: **Glycoproteinosis (18):** Alpha-Mannosidosis (16): Aspartylglucosaminuria (AGU) (4), Fucosidosis (Type I and II) (3), Galactosialidosis (Cathepsin A Mutation) (1), Mucolipidosis Type I (Sialidosis) (1), Mucolipidosis Type II (i-Cell Disease) (2), Mucolipidosis Type IV (2)., α-N-acetylgalactosaminidase Deficiency (Schindler Disease Type I) (1). **Lysosomal storage disorders of lipid metabolism and transport (76):** Acid sphingomyelinase deficiency (Niemann Pick A) (3), Gaucher Type 2 (2), Gaucher Type 3 (12), Globoid Cell Leukodystrophy (Krabbe Disease) (9), GM1 gangliosidosis (7), GM2 gangliosidosis – non-specified (10), GM2 gangliosidosis (Tay-Sachs disease) (1), Metachromatic leukodystrophy (27), Niemann-Pick C (19), Saposin C Deficiency (1). **Mucopolysaccharidoses (79):** MPS I Hurler Syndrome (25). MPS II Hunter Syndrome (23), MPS III Sanfilippo Syndrome (33), MPS VII Sly Syndrome (6). **Other lysosomal diseases (10):** Neuronal Ceroid Lipofuscinoses (Batten Disease) (10). **Other disorders of lipid metabolism and transport (5):** Abetalipoproteinaemia (1), Cerebrotendinous Xanthomatosis (4). **Disorders of amino acid and other organic acid metabolism (6):** Canavan Disease (5), Sulfite/Sulphite Oxidase Deficiency (1). **Vitamin-responsive inherited metabolic disorders (6):** Cobalamin C Disease/Deficiency (1), Molybdenum Cofactor Deficiency (5). **Disorders of mineral absorption and transport (12):** Wilson disease (12). **Peroxisomal disease (16):** X-linked adrenoleukodystrophy (13), Zellweger Spectrum Disorder (4). **Mitochondrial disorders (12):** Kearns-Sayre syndrome (3), Leigh disease (5), MELAS (8), POLG-related disease (1). **Other inherited metabolic disorders (11):** Congenital disorders of glycosylation (8), Pyruvate dehydrogenase deficiency (3). **Leukodystrophies not otherwise categorized (4):** Alexander Disease (Type I) (1), Pelizaeus Merzbacher Disease (2), Vanishing White Matter Disease (1). **Neurodegeneration with brain iron accumulation (5):** Beta propeller protein associated neurodegeneration (BPAN) (1), Coenzyme A synthase protein-associated neurodegeneration (COASY) (1), Pantothenate kinase-associated neurodegeneration (PKAN) (5). **Neurodegenerative diseases not otherwise categorized (22):** Cockayne Syndrome (1), Giant axonal neuropathy (1), Huntington's Disease (Juvenile Form) (1), Infantile Neuroaxonal Dystrophy (1), Rett Syndrome (18).

Across the 262 studies, 49 individual childhood dementia disorders were observed, with Sanfilippo syndrome (MPS III) and metachromatic leukodystrophy, noted as the most common conditions (Fig. 2). Of the 72 childhood dementia disorders examined, twenty-three conditions had an absence of clinical trial activity incorporating biological measures. An observational study design was adopted by 64 studies and an interventional model by 198 clinical trials.

The latter included the following approaches: (6/198, 3 ​%) cell therapy, (25/198, 13 ​%) gene therapy, (10/198, 5 ​%) that combined cell and gene therapy, (67/198, 34 ​%) enzyme replacement therapy, (77/198, 39 ​%) small molecule drugs, (12/198, 6 ​%) dietary modification/supplement, and (6/198, 3 ​%) others (e.g., behavioural intervention). Interventional studies were undertaken across either individual or multiple phases of clinical research, with Phase 1 (n ​= ​76), Phase 2 (n ​= ​122), Phase 3 (n ​= ​49) and Phase 4 (n ​= ​14) approaches. The estimated enrolment targets for observational studies totalled 11,243 participants, with variability of patient recruitment between studies: mean 175 (SD 300), median 42 (IQR 80) and a range 0–1000 participants. Enrolments were lower for interventional studies: total 5,635, mean 28 (SD 43), median 18 (IQR 23) and range 0–400 participants. The sharing of individual participant data in clinical trial databases was planned in 8.4 ​% (22/262) of all studies, while 71.4 ​% (187/262) intended to withhold this data (Supplementary Table 1). The assessment of circulating biomarkers was mostly conducted on blood constituents (serum n ​= ​65, plasma n ​= ​57, whole blood n ​= ​6, peripheral blood mononuclear cells n ​= ​13), cerebrospinal fluid samples (n ​= ​94), and urine (n ​= ​96). Of the 262 studies, 160 were completed at the time of censoring, and of these, 73 (45.6 ​%) had reported results either by publication (51/262, 19 ​%) or in the trial registry databases, comprising 4/36 (11.4 ​%) observational studies and 69/125 (55.2 ​%) interventional studies.

Three key themes were identified across the assessed clinical trials. These were *‘disease-related biomarkers’* associated with 1) the primary pathophysiological pathway or 2) relevant downstream pathogenic events; and 3) *‘drug-related biomarkers’* assessing the pharmacokinetics, safety, and/or tolerability of an investigational product.

#### Disease-related biomarkers – primary pathophysiological pathway

**Fig. 3:**
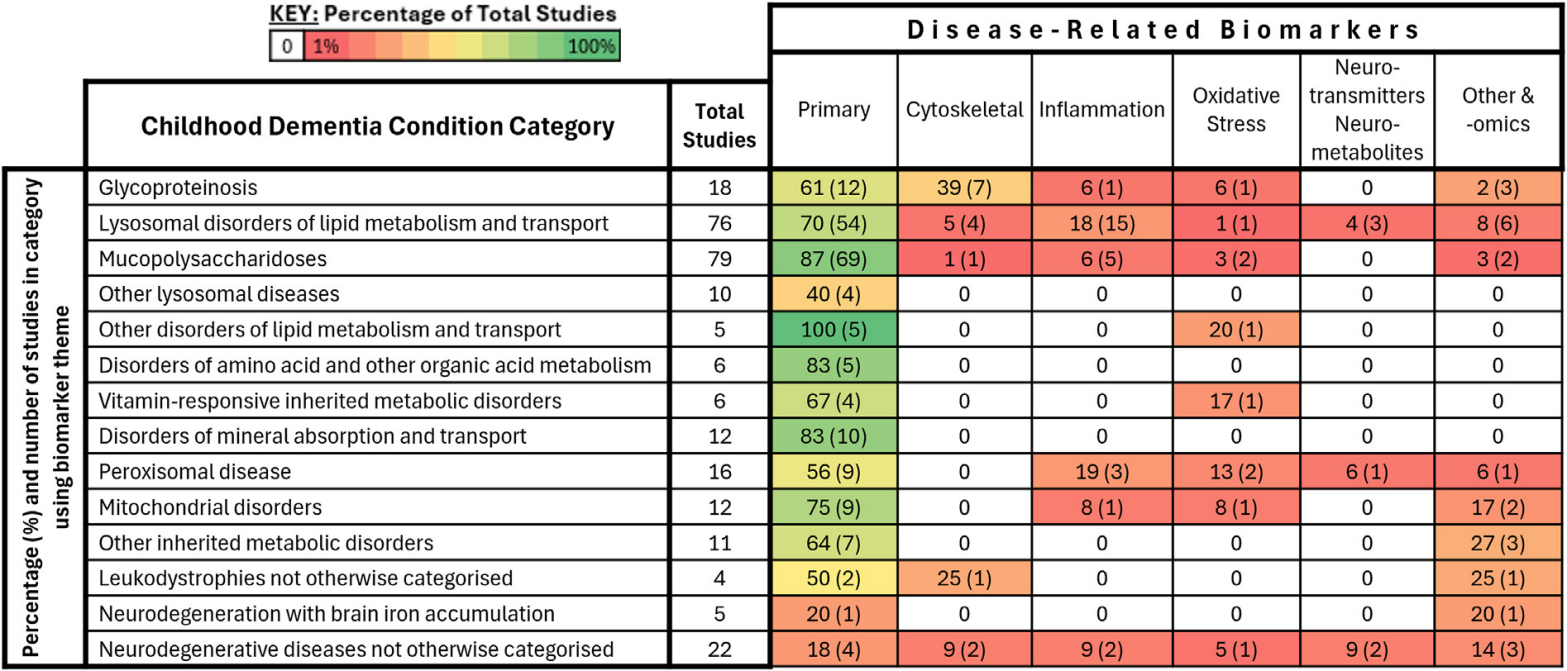
**Summation of disease-related biomarker use across childhood dementia condition categories.** The ‘Total Studies’ refers to the number of studies which examined conditions in that category. Some studies examined multiple categories. The percentage is calculated as the number of times that biomarker theme was used in a study of that condition category, given in parentheses, divided by ‘Total Studies’ for that condition category.

Biomarkers connected to the **primary pathophysiological pathways** had their targeted use reported in 185/262 (70.8 ​%) studies. There were 102 individual biomarker measures identified and associated with the affected metabolic pathways of specific diseases (Table 1). These biomarkers were used more frequently as primary or secondary outcome measures in studies that investigated child dementias broadly classified as ‘inborn errors of metabolism’ (76.7 ​%, 178/232), and much less frequently in studies of conditions otherwise classified (22.6 ​%, 7/31) (Fig. 3).

**Table 1:** Disease-related circulating biomarkers connected to the primary pathophysiologic pathway listed as outcome measures in clinical studies in childhood dementia disorders.

Category	Biomarker	Associated diseases
Lysosomal disorders of lipid metabolism and transport	Glycolipids	Gaucher disease type 2, Gaucher disease type 3
	Lyso-Gb1, Lyso GL1, Lyso-GM3	Gaucher disease type 3
	GM1 ganglioside, β-galactosidase activity and substrates: dp5, A2G2’, glycosaminoglycans (keratan sulfate, heparan sulfate, dermatan sulfate, chondroitin-6-sulfate)	GM1 gangliosidosis
	GM2 ganglioside	GM2 gangliosidosis (Sandhoff disease, Tay Sachs disease)
	GL-1 (glucocerebroside),	GM2 gangliosidosis (Sandhoff disease, Tay Sachs disease), Gaucher disease type 3, Saposin C deficiency
	Hexosaminidase (Hex A and Hex B) enzyme activity and levels	GM1 gangliosidosis, GM2 gangliosidosis (Sandhoff disease, Tay Sachs disease)
	Arylsulfatase activity, sulfatide	Metachromatic leukodystrophy (MLD)
	Sphingomyelin, Lyso-sphingomyelin, acid sphingomyelinase activity Total cholesterol, high density lipoprotein (HDL) and low-density lipoprotein (LDL) cholesterol.	Acid sphingomyelinase deficiency (Niemann pick type A)
	Cholesterol precursors: Lanosterol, lathosterol, desmosterol Cholesterol metabolites: 24S hydroxycholestrol, Cholesterol metabolites/bile acid precursors (4b-, 24S-, 25-, 27- hydroxycholesterol) Cholesterol esterification: Cholestane-triol, 7-ketocholesterol Oxysterols: 24S-hydroxycholesterol, 25-hydroxycholesterol, and 27-hydroxycholesterol Un-esterified cholesterol Lyso-SM-509 NPC1 protein N-palmitoyl-O-phosphocholineserine (PPCS) Cholestane-3β Bile acid B (5α) C-Triol (6β-triol)	Niemann pick type C (NPC)
	Psychosine, GALC enzyme activity	Globoid cell leukodystrophy (Krabbe disease)
Mucopolysaccharidoses	Glycosaminoglycans e.g. Heparan sulfate, dermatan sulfate, chondroitin sulfate	Hunter, Hurler, Sanfillipo syndrome, Sly syndrome
	SGSH enzyme activity, NAGLU enzyme activity	Sanfillipo syndrome
Glycoproteinosis	Mannose rich oligosaccharides	Alpha-mannosidosis
	Glycoasparagines, leukocyte AGA enzyme activity	Aspartylglucosaminuria (AGU)
Other lysosomal diseases	Granular osmophilic deposits	Neuronal ceroid lipofuscinosis (Batten disease)
Other disorders of lipid metabolism and transport	Cholestanol, lipid, lipoproteins	Cerebrotendinous xanthomatosis
	Carotenoids	Abetalipoproteinemia
Disorders of amino acid other organic acid metabolism	N-acetyl aspartate (NAA), acetate	Canavan disease
Vitamin-responsive inborn errors of metabolism	S-sulfocysteine (SSC), sulfite, xanthine, uric acid	Molybdenum cofactor deficiency, Sulfite oxidase deficiency
Disorders of mineral absorption and transport	Caeruloplasmin, copper, non-caeruloplasmin bound copper	Wilson disease
Peroxisomal disease	Very long chain fatty acids (VLCFA): C26, C24, C22, C26:C22	X-linked adrenoleukodystrophy
	Very long chain fatty acids (VLCFA): Phytanic acid, Plasmalogen, Pipecolic acid	Zellweger
Mitochondrial disorders	Glutathione, glutathione disulfide, glutathione cycle biomarkers	Leigh disease, Kearns-Sayre, MELAS, POLG-related disease, Pearson syndrome
	Lactate	Leigh disease, MELAS
	Lactic acid	Kearns-Sayre
	Acetoacetate	Leigh disease
	Beta-hydroxybutyrate	Leigh disease
	Creatine kinase, amino acids (arginine, ornithine, citrulline	MELAS
	Nitric oxide	MELAS
Other inborn errors of metabolism	Transferrin glyco isoforms, antithrombin III, coagulation factor IX and XI, IGFBP3 and TSH Sorbitol, mannitol, Antithrombin III (ATIII). Liver transaminases. Transferrin glycosylation PMM2 biomarker carbohydrate deficient transferrin	Congenital disorders of glycosylation
	Lactate, pyruvate, β-hydroxybutyrate (β-OHB)	Pyruvate dehydrogenase deficiency
Leukodystrophies not otherwise categorized	Glial fibrillary acidic protein (GFAP)	Alexander disease
	Proteolipid protein 1 (PLP1)	Pelizaeus-Merzbacher disease
Neurodegeneration with brain iron accumulation	Hemochrome	BPAN, NBIA, PKAN
	Coenzyme-A synthetase mRNA	COASY
Diseases not otherwise categorized	Global DNA methylation Metabolic markers of methylation: Methionine, homocysteine, SAM, SAH.	Rett syndrome

**ABBREVIATIONS:** β-OHB: β-hydroxybutyrate. **A2G2**: NA2 glycan. **AGA**: Aspartylglucosaminidase. **AGU:** Aspartylglucosaminuria. **ATIII**: Antithrombin III. **BPAN**: Beta-propeller protein-associated neurodegeneration. **COASY**: Coenzyme A Synthase. **DNA:** deoxyribonucleic acid. **Dp5:** Cell death-promoting gene 5. **GALC:** galactocerebrosidase. **Gb1/GL** galactocerebrosidase. **Gb lyso-GM3**: Sialyllactosyl sphingosine. **GFAP**: Glial Fibrillary Acidic Protein. **GM1**: monosialotetrahexosyl-ganglioside 1. **HDL-C**: High density lipid cholesterol. **IGFBP3**: Insulin-like growth factor-binding protein 3. **LDL-C**: Low density lipid cholesterol. **Lyso-SM**: Lyso-sphingomyelin. **MELAS:** Mitochondrial Encephalopathy, Lactic Acidosis, and Stroke-like episodes. **MLD:** metachromatic leukodystrophy. **NPC:** Niemann-Pick Disease Type C. **NAA**: N-acetyl aspartate. **NAGLU:** α-N-acetylglucosaminidase. **NBIA**: Neurodegeneration with Brain Iron Accumulation. **PKAN**: pantothenate kinase-associated neurodegeneration. **PMM2:** phosphomannomutase 2. **POLG:** polymerase gamma. **PLP1:** Proteolipid Protein 1. **PPCS:** N-palmitoyl-O-phosphocholineserine**. (m)RNA**: messenger ribonucleic acid. SAH: S-adenosylhomocysteine. **SAM**: S-Adenosyl methionine. SGSH: N-sulphoglucosamine sulphohydrolase. **SSC**: S-sulfocysteine. **TSH:** Thyroid-stimulating hormone. **VLCFA:** Very Long Chain Fatty Acid.

Results for these were reported, through trial registry or publication, in (n ​= ​51/111; 45.9 ​%) of studies completed at censoring date (Supplementary Table 2).

Among these 51 studies, biomarkers fulfilled 94 roles, the most common of which was to assess pharmacodynamic response (n ​= ​58/94, 62 ​%). These biomarkers were less frequently used to monitor disease status, progression, or to serve as surrogate endpoints for a clinical trial (n ​= ​26/94, 28 ​%). Furthermore, few were used for diagnostic, sub-type stratification, or prognostic purposes (n ​= ​10/94, 10 ​%). Among these studies, biomarkers were rarely used as primary outcome endpoints (n ​= ​17/51, 33 ​%), and then mostly in observational or phase 1/2 trials (n ​= ​13/17, 76 ​%) (Supplementary Table 2).

The following examples illustrate the utility of these biomarkers in studies of childhood dementias. **Bolded** words relate to the biomarkers’ classified roles [11].

##### Role in NPC therapeutic intervention development

Niemann-Pick type C (NPC) is a lysosomal disorder of lipid and cholesterol metabolism [12]. Observational studies **monitored** PBMC HSP70 and unesterified cholesterol levels, along with serum cholestane-triol – the latter two were elevated as measures of lipid burden [13]. The drug Arimoclomol activates the heat shock response, which includes HSP70 – a protein associated with neuroprotection and proper cholesterol-related functioning of the NPC1 protein [12,14,15]. Furthermore, interventional trials of this drug used these biomarkers. Serum cholestane-triol was used to helped **diagnose** participants suitable for enrolment, while PBMC HSP70 and unesterified cholesterol, serum-cholestane triol, and plasma lyso-sphingomyelin-509 (lyso-SM-509) helped assess **efficacy** by measuring **pharmacodynamic response**. Compared to a placebo, the treatment slowed PBMC unesterified cholesterol increase, increased PBMC HSP70, and reduced levels of serum cholestane-triol and plasma lyso-SM-509. Lower plasma lyso-SM-509 is correlated with reduced disease severity and later onset of neurological disease. Collectively, these biomarkers were interpreted as evidence of target engagement and biological activity through heat shock response activation [12]. Despite this biomarker evidence, the drug was not fully approved for treatment of NPC, since the primary endpoint measure was instead based on clinical assessments that required further validation [16,17].

##### Role in MPS

In mucopolysaccharidoses (MPS I-III) enzymatic changes cause glycosaminoglycans such as heparan/dermatan sulfate to accumulate in organs and the central nervous system. Accordingly, trials used their levels in CSF, plasma, or urine as biomarkers of **pharmacodynamic response** (mostly to enzyme replacement or gene therapies) with a reduction in levels representing treatment benefit [[18], [19], [20], [21], [22], [23]]. Observational studies also used glycosaminoglycans as **monitoring** biomarkers to indicate disease status, but due to their stable levels over time they were concluded to be ineffective at assessing disease progression or severity [[24], [25], [26]].

##### Role in alpha-mannosidosis

In alpha-mannosidosis, deficient alpha-mannosidase enzyme activity in the lysosomes causes the toxic accumulation of mannose-rich oligosaccharides [27]. **Monitoring** their urinary and CSF levels in natural history observational studies found that their elevation was correlated with disease status and severity [28]. This enabled their levels in CSF, serum, and urine to be used as **pharmacodynamic** and **surrogate endpoint** markers in interventional trials, which correlated their decrease with clinical improvements [[29], [30], [31], [32], [33]].

In studies of the peroxisomal diseases X-linked adrenoleukodystrophy and Zellweger syndrome, deficiency or dysfunction of the peroxisome organelle was shown to lead the accumulation of Very Long Chain Fatty Acids (VLCFAs) or impaired lipid synthesis. The level of VLCFA in blood and plasma was used as **diagnostic** biomarker for inclusion (when elevated) and to assess **pharmacodynamic** response to treatment [34,35].

#### Disease-related biomarkers – relevant down-stream pathogenic events

Biomarkers associated with **relevant down-stream pathogenic events** were listed as secondary or exploratory measures in 61/262 (23.3 ​%) studies. There were 51 individual biomarker measures specified and five subcategories were distinguished: (i) cytoskeletal proteins (15/61, 24.6 ​%), (ii) cytokines, chemokines, and other inflammatory mediators (24/61, 39.3 ​%), (iii) surrogates of oxidative stress and antioxidants (8/61, 13.1 ​%), (iv) neurotransmitters or related neuro-metabolites (5/61, 8.2 ​%), and (v) other exploratory, including those that were non-targeted and used in a discovery approach (20/61, 32.8 ​%) (Table 2). Results for these were reported, via trial registry or publication, in 32.5 ​% (14/40) of studies completed at censoring date, with details on the biomarker roles and study findings summarized in Supplementary Table 3.

**Table 2:** Disease-related circulating biomarkers associated with downstream pathogenic processes listed as outcome measures in clinical studies in childhood dementia disorders.

Category	Biomarker	Associated diseases
Cytoskeletal proteins	Neurofilament (NfL, NEFL)	Alpha-mannosidosis, Cockayne syndrome, GM1 gangliosidosis, Juvenile Huntington's disease, metachromatic leukodystrophy, Rett syndrome
	Glial fibrillary acid protein (GFAP)	Alexander disease, alpha-mannosidosis, Cockayne syndrome, metachromatic leukodystrophy, Niemann-pick type C
	Tau	Alpha-mannosidosis, Cockayne syndrome, metachromatic leukodystrophy, Niemann-pick type C, Sanfilippo syndrome
Cytokines, chemokines, and other inflammatory markers	Tumor Necrosis factor alpha (TNF-α)	Hurler syndrome, GM1 gangliosidosis
	Interferon gamma	Hurler syndrome, GM1 gangliosidosis
	Interleukin 1 beta	Hurler syndrome, GM1 gangliosidosis
	Interleukin 2	Hurler syndrome, GM1 gangliosidosis
	Interleukin 8	GM1 gangliosidosis, hurler, X-linked ALD
	Pro-inflammatory and anti-inflammatory cytokine levels	Rett syndrome, Sanfilippo, X-linked ALD
	Pro-inflammatory cytokine panel	GM1 gangliosidosis
	Macrophage inflammatory protein-1 beta	X-linked ALD
	Monocyte chemoattractant protein-1	X-linked ALD
	Chemokine ligand 5 (RANTES)	GM1 gangliosidosis, hurler syndrome
	Macrophage inflammatory protein 1 alpha	GM1 gangliosidosis, hurler syndrome
	Chitotriosidase	X-linked ALD, Gaucher disease type 3, acid sphingomyelinase deficiency (niemann pick type A), metachromatic leukodystrophy
	Arachidonic acid metabolites	X-linked ALD
	Macrophage functionality	Metachromatic leukodystrophy
	Migration inhibitory factor (MIF)	Niemann-pick C
	CC Motif chemokine Ligand 18 (CCL18)	Acid sphingomyelinase deficiency (niemann pick type A), Gaucher disease type 3, niemann pick type C
	Complement 3A and 5A	Gaucher disease type 2, niemann pick type C
	Complement C5b-9	Gaucher disease type 2, niemann pick type C
Oxidative stress	Glutathione and relevant glutathione metabolites glutathione peroxidase (GPx),	Abetalipoproteinemia, Cobalamin C disease, hurler syndrome, Kearns-Sayre syndrome, Rett syndrome, X-linked ALD,
	Biomarkers of redox state	Cobalamin C disease, hurler syndrome
	Antioxidant enzymes	Cobalamin C disease
	Antioxidant N-acetylcysteine	Alpha-mannosidosis, Aspartylglucosaminuria, fucosidosis, hurler syndrome, Krabbe disease, Niemann-pick type C, Sly syndrome,
	Superoxide dismutase	Abetalipoproteinemia, hurler syndrome
	8-isoprostane	Hurler syndrome
	Thiobarbituric acid reactive substances	Hurler syndrome
	Catalase	Hurler syndrome
	Carbonyl	X-linked ALD
	4-hydroxynonenal (4-HNE)	Hurler syndrome, X-linked ALD
	Malondialdehyde, vitamin C, oxidized cholesterol, F2-isoprostanes.	Abetalipoproteinemia
Neurotransmitters and related metabolites	Glutamate	Rett syndrome
	Gamma-aminobutyric acid (GABA)	Rett syndrome
	N-acetyl aspartate (NAA)	Metachromatic leukodystrophy
Other exploratory biomarkers (including non-targeted -omics)^a^	Mass Spectrometry-based approach	Alpha-mannosidosis, hurler syndrome, Globoid cell Leukodystrophy//Krabbe disease, Mucolipidosis type II, Sly syndrome
	Metabolomic Macrophase metabolic	Congenital disorders of glycosylation, Gaucher disease type 3, Kearns-Sayre syndrome, metachromatic leukodystrophy, NBIA, X-linked ALD
	Methylomic	Gaucher disease type 3, Rett syndrome
	Transcriptomic Macrophage transcriptomic	Gaucher disease type 3, X-linked ALD
	Molecular and biochemical markers	Congenital disorders of glycosylation, Mucolipidosis type II
	CSF/Serum albumin index	Metachromatic leukodystrophy
	Ubiquitin C-terminal hydrolase (UCHL1)	Cockayne syndrome
	Brain derived Neurotrophic factor (BDNF)	Rett syndrome
	Heat shock protein 70 (Hsp70)	Niemann-pick Type C

**ABBREVIATIONS: 4-HNE**: 4-hydroxynonenal. **ALD**: adrenoleukodystrophy. **BDNF**: Brain Derived Neurotrophic Factor **CCL18**: Cysteine-Cysteine Motif Chemokine Ligand 18. **CSF**: Cerebrospinal fluid. **GABA**: Gamma-aminobutyric acid. **GM1**: monosialotetrahexosyl-ganglioside 1. **GPx**: Glutathione peroxidase. HSP70: Heat Shock Protein 70. **MIF**: Migration Inhibitory Factor. **NAA**: N-acetyl aspartate. **NBIA**: Neurodegeneration with Brain Iron Accumulation. **NEFL/NfL**: Neurofilament. **RANTES**: Regulated upon activation. Normal T Cell expressed and presumably Secreted. **TNF-α**: Tumour Necrosis Factor Alpha. **UCHL1**: Ubiquitin C-terminal hydrolase.

a High throughput molecular technologies and a discovery-based approach were used in this category to identify potential transcriptomic, metabolomic and methylomic surrogates of disease.

*Cytoskeletal proteins* were reported as biomarkers in 15 studies, and used for purposes of disease **monitoring**, exploratory **diagnostic stratification**, **prognosis**, and to assess **pharmacodynamic response**. In the latter role, for studies of metachromatic leukodystrophy and Niemann-Pick type C, cytoskeletal proteins, such as CSF tau, GFAP, and neurofilament light chain (NfL), were elevated at baseline and decreased with treatment, correlating with clinical improvements [36,37]. In contrast, in Rett syndrome CSF and serum NfL was static pre- and post-treatment and did not correlate with changes in clinical scores [38].

For a study of MPS IIIA, CSF tau was similarly leveraged for its capacity to **monitor** therapeutic intervention. However, CSF tau in this condition did not yield **prognostic** value as despite elevation relative to healthy controls no relationship was established with disease progression [24].

In alpha-mannosidosis studies of intravenous enzyme replacement therapy, overall, the combination of primary pathophysiological metabolites and secondary downstream cytoskeletal proteins indicated a **pharmacodynamic response** systemically but a lack of biological activity in the CNS [31,32]. A **prognostic** role for these cytoskeletal proteins was also explored, since they were elevated compared to healthy controls and negatively correlated with cognitive function, thus having potential relevance to treatment planning [39].

*Biomarkers of inflammation*, encompassing chemokines and cytokines, were incorporated in 24 clinical studies. Their use as markers of **pharmacodynamic response** was particularly observed in interventional studies of Gaucher type 3, Niemann-Pick type C, and MPS III (Sanfilippo). In a Gaucher 3 study, blood levels of chitotriosidase (CHIT1) and CCL18 were used to assess pharmacodynamic response, and **diagnostically** for recruitment [40]. In a Niemann-Pick type 3 study, CSF levels of TNF-α and GFAP were intended for measurement of pharmacodynamic response [37]. In MPS III, a panel of CSF and plasma chemokines and cytokines were examined [41]. In an observational study of NPC, CSF CCL18 was used for **monitoring**, and noted for **prognostic** potential [42].

*Oxidative stress-related* biomarkers, including antioxidants, were identified in 8 studies. Results were reported for a study of X-ALD, in which whole blood glutathione (GSH) levels were evaluated as biomarkers of therapeutic efficacy to vitamin D supplementation, with static levels indicating a lack of **pharmacodynamic response** [43]. *Neurotransmitters and related neurometabolites* were identified as circulating biological markers in 5 of the studies reviewed here, with none reporting the results of their use. *Exploratory biomarkers* were also reported, along with those that did not map clearly to the preceding ‘downstream’ categories, such as neuroprotective markers, in 20 studies. These included studies using untargeted ‘-omics’ approaches (e.g., metabolomics, methylomics, transcriptomics) and studies aimed at identifying novel biomarkers. In a Rett syndrome study, methylomics showed increase in global RNA methylation in **response** to creatine monohydrate supplementation [44]. Other Rett syndrome studies utilised Brain Derived Neurotrophic Factor (BDNF), a molecule associated with neuroprotection [45,46]. In a trial of the drug Fingolimod in children with Rett syndrome, CSF BDNF levels were higher at baseline and were associated with better clinical scores [38]. Another trial reported lower serum BDNF for Rett patients compared to the general population, and showed an increase in **response** to enriched environment intervention, alongside gains in gross motor skills [47]. In a study of MLD, an index of the ratio of albumin in CSF to serum, an indicator of impaired blood-brain barrier integrity, was used to evaluate **pharmacodynamic response**. A heightened baseline ratio was correlated with higher CSF levels of disease-related sulfatides, with a decrease post-treatment considered an improvement. The published results also considered its potential as a **prognostic** marker of disease severity, albeit with cautious use due to variable accuracy [36]. Collectively, these results show the limited integration of potential biomarkers of neurodegeneration, neuroinflammation, or oxidative stress into clinical trials involving childhood dementias (Fig. 3).

### Drug-related biomarkers

The nature and range of safety and tolerability biomarkers were similar across studies and comprised standard clinical laboratory assessments (hematology, hepatic aminotransferases, bilirubin, creatinine, thyroid function) and urinalysis. These were detailed in 97/262 (37 ​%) studies and were also utilised to identify participants with safety risks for whom therapies should not be initiated, with published results available for 34/60 (56.7 ​%) completed studies. In addition, the assessment of specific neutralizing antibodies was conducted in 47/262 (17.9 ​%) studies. This was employed, typically as a predictive biomarker to identify potential non-responders alongside other pharmacodynamic disease-related biomarkers, for 31/67 (46.3 ​%) of enzyme replacement therapy studies, for 5/25 (20 ​%) of gene transfer therapy studies, and for 6/10 (60 ​%) of combined cell and gene therapy studies. Markers of pharmacokinetic efficacy, including generic pharmacokinetic parameters (maximal concentration, half-life, area under the curve, distribution, and clearance) were listed as outcome measures in 112/262 (42.7 ​%) studies and published results were available for 37/72 (51.4 ​%) completed studies.

## Discussion

Technological advancements, particularly in cell and genetic therapies, offer new opportunities for childhood dementia disorders, requiring careful planning of clinical trial design and readiness for small populations [48,49]. In this scoping review, we synthesized findings from a wide range of clinical studies across nearly fifty individual childhood dementia disorders.

The results showed the utility and varied role of a range of circulating biomarkers, aligning with and representing the complex pathways within childhood dementia. Though heterogenous in genotype and clinical phenotype, the results of this study denoted shared pathogenic primary processes and downstream sequalae. The findings highlight the ability of these biomarkers to address gaps in prognostication, prediction of disease evolution and therapeutic efficacy and safety amongst affected individuals across the disease spectrum.

Thus far, clinical trials have focused on key metabolites associated with individual metabolic disorders, and there have been few studies integrating potential biomarkers of neurodegeneration, neuroinflammation or oxidative stress. Pharmacodynamic and monitoring biomarkers have served different purposes, including identification of drug target engagement and measurement of downstream treatment effects, and been crucial to informing decisions regarding moving forward with the development of disease modifying therapies.

This review has identified for the first time, common platforms for biomarker development in childhood dementias, including neuroinflammation [50,51], oxidative stress [52], neuronal dysfunction [53], altered lipid homeostasis, disturbances in DNA and RNA biology [54] and aberrant protein dynamics [55]. These biomarkers are potential targets for additional pre-clinical and exploratory studies, to progress understanding of their scope in developing a unifying platform of biomarkers that can be leveraged across the spectrum of conditions that encompass childhood dementia.

Whilst the scoping review has identified key targets for biomarker discovery, leveraging interrogation of studies in other non-dementia related neurological conditions will also be vital to providing a comprehensive view of surrogates of disease and treatment. Focusing on the major theme of neuroinflammation mediated neurodegeneration as emphasised in the results of this review, CSF metabolites in the tryptophan-kynurenine, nitric oxide pathways and neopterin are emerging as useful diagnostic and monitoring biomarkers of neuroinflammation. As such, these warrant assessment in childhood dementia to identify and monitor potentially treatable processes that can influence neurodegeneration [56,57].

Previous studies have also established the role of major lipid pathways in the modulation of oxidative stress and inflammatory responses, including release of arachidonic acid, docosahexaenoic acid, and linoleic acid [57,58]. This is of relevance in childhood dementia as altered metabolism of ceramide, cholesterol, phosphatidylcholine, sphingomyelin, and sulphatides, (the most abundant lipid classes in the CNS) occurs in a number of childhood dementia conditions [58].

Neurofilaments, structural cytoskeletal proteins that are released following axonal injury or neuronal loss, are being considered as prognostic or monitoring biomarkers in a number of neurodegenerative diseases, including Alzheimer's disease, frontotemporal dementia, multiple sclerosis, Parkinson's disease, and spinal muscular atrophy [[59], [60], [61], [62]]. Among these diseases, higher neurofilament levels have been found to reflect disease activity and suppression of levels have been associated with the clinical effectiveness of therapies [63]. Taken together these highlight the need for integrated biological analyses of metabolomics, lipidomics, and proteomics to further define and monitor neuroinflammation mediated neurodegeneration.

Linked to the themes of oxidative injury as identified in this review, surrogates of mitochondrial dysfunction are an ideal target to evaluate from a biomarker perspective for monitoring purposes but with a dual role to reveal novel therapeutic targets. These include growth differentiation factor 15 (GDF-15), fibroblast growth factor-21 (FGF-21), microRNA, mitochondrial membrane potential, and oxidative phosphorylation (OXPHOS) activity in blood [64]. Further studies have incorporated biomarkers to inform clinical care, using integrated multi-omics approaches to diagnose a unique cohort in Methylmalonic aciduria (MMA). Dysregulation of the tricarboxylic acid cycle and its replenishment by glutamine was highlighted as a potential therapeutic intervention point in MMA [65].

Whilst this scoping review is the first to establish a comprehensive overview of the current biomarker landscape in childhood dementia disorders, several limitations warrant discussion. Variations in trial design, protocol changes, and secondary analyses may preclude generalisation and applicability to a real-world population. Although a broad spectrum of metabolic changes has been reported in childhood dementia as seen in this review, translatable biomarkers that are useful in clinical practice have not yet been reported. There is a lack of consistency and further validation in identified biomarkers across independent studies is required. A major challenge in translational studies is the subsequent validation of biomarkers through absolute quantification to evaluate the sensitivity and specificity in larger populations and longitudinal studies. The heterogeneity of phenotypes in childhood dementia requires the development of standardized and patient relevant outcome measures, to aid development of a representative biomarker repertoire. Limitations of the data available in this field includes the lack of commitment to reporting study findings consistently, drawing attention to the need for transparency of data reporting and sharing.

The number of childhood dementia disorders within the childhood dementia knowledgebase continues to increase, however with ultrarare diseases having limited clinical trial opportunities relative to their epidemiology, our findings are likely to be representative. Limitations of the current study include aggregation of data from studies registered on the clinical trials databases in English; therefore, informative studies not listed or published in other languages were possibly missed.

Future directions should account for and address the knowledge gaps including assay and biomarker validation, alongside establishing age-matched normative reference ranges. The composition and metabolic activity in human CSF, plasma and microbiota are profoundly intertwined with human health and disease status. CSF provides unique insights into brain function without the need for brain tissue biopsy. However, given the less invasive and accessible nature of blood, it provides an important reflection of the metabolic changes and the role of the blood brain barrier. The simultaneous analysis of CSF and blood would be beneficial in understanding the origins of metabolic changes and whether the changes are secondary to blood brain barrier disruptions.

Whilst the scoping review identified emergent technologies for biomarker interrogation, the expansion of omic technologies (such as transcriptomics, proteomics, metabolomics) is a powerful tool to fingerprint biological profiles, building our knowledge of molecular networks associated with immune and cellular functions implicated in disease onset, severity and progression. The combination of omics techniques as a discovery-driven approach are highly complementary and largely untapped. Targeted approaches create a translational process to quantify and validate discriminative findings from untargeted studies to assess their sensitivity, specificity and reproducibility as biomarkers.

Lastly, therapeutic development and regulatory approvals are increasingly founded on biomarker discovery programs. The integration of biomarkers in clinical development, approval and practice is recognized by the United States Federal Drug Administration, and the BEST resource was created to consistently define and describe the diverse roles of biomarkers [11]. Surrogate biomarkers are further characterized by the level of clinical substantiation and high quality, sensitive, robust trials, increase the efficiency of patient-centred therapeutic access [[66], [67], [68]]. Within childhood dementias which can have variable natural histories, meaningful clinical endpoints may not be viably obtained in the timespan of the trial, which stall treatment development. Biomarkers that are used as surrogate endpoints can generate quantifiable benefits in clinical trial performance and accelerate the drug delivery process.

Additional recommendations for further research include, stratifying clinical cohorts based on shared molecular drug targets, to increase opportunities for engagement in research and to investigate and validate multiple relevant biomarkers in master protocols. Not only will this improve the power of data to inform treatment development, but also ensure that risks and benefits are rationalised for children with dementia [69].

## Data sharing

Data underlying the results reported in this manuscript can be made available to suitably qualified researchers through reasonable request. Applicants should apply between one and 12 months after the manuscript has been published in print. The data request should then be sent to the corresponding author, and data will be shared with a signed data access agreement.

## Author contributions

MAF and ADS formulated the concept of the manuscript. KE established and curated the Childhood Dementia Knowledgebase that identified the conditions in this study. AD, JD, and JB performed the study search and selection, data collection, coding, and analyses. MF contributed to study selection, coding, and analyses. MF and AD wrote the first and subsequent drafts of the manuscript, including constructing all tables and figures. All authors contributed to manuscript revision, read, and approved the final manuscript.

## Funding statement

This study received grant support from the Medical Research Future Fund (Improving health outcomes by identifying biomarkers to delineate common mechanistic pathways and to monitor therapeutic effect of clinical trials in childhood dementia Grant ID: 2022/MRF2023012).

## Declaration of competing interest

Kristina Elvidge is the Head of Research at the Childhood Dementia Initiative. All other authors declared no potential conflicts of interest with respect to the research, authorship, and/or publication of this article.

## Appendices

### Appendix 1 List of childhood dementia conditions included in search and alternative search terms (n ​= ​72)

AARS2-related leukodystrophy

Abetalipoproteinaemia

Abetalipoproteinemia

Acid sphingomyelinase deficiency OR Niemann-Pick A

Alexander Disease

Alpers-Huttenlocher syndrome

Alpha-Mannosidosis

Aspartylglucosaminuria

Batten disease OR CLN1/CLN2/CLN3/CLN5/CLN6/CLN7/CLN8/CLN10 OR Neuronal ceroid lipofuscinosis 3

Biotin-Thiamine-Responsive Basal Ganglia Disease

Biotinidase deficiency

BPAN OR NBIA

Canavan disease

Cerebral Folate Deficiency

Cerebrotendinous xanthomatosis

Cobalamin C disease

Cockayne syndrome

Combined Saposin Deficiency

Congenital disorders of glycosylation

CoPAN OR COASY OR NBIA

FAHN OR NBIA

Farber Disease

Fucosidosis

Galactosialidosis

Gaucher disease type 2

Gaucher disease type 3 OR neuronopathic Gaucher Disease

Giant axonal neuropathy

Glutathione Synthetase Deficiency

Glycine Encephalopathy

GM1 gangliosidosis

GM2 gangliosidosis OR Tay-Sachs Disease OR Sandhoff Disease OR AB Variant

Holocarboxylase synthetase deficiency

Hunter syndrome OR Mucopolysaccaridosis type 2 OR MPS2

Hurler syndrome OR Mucopolysaccharidosis type I OR MPS1

Infantile neuroaxonal dystrophy OR INAD OR NBIA

Juvenile Huntington's disease

Juvenile Parkinson's Disease

Kearns-Sayre Syndrome

Krabbe Disease

Kufor-Rakeb Syndrome OR NBIA

Lafora disease

Leigh Disease

MECP2 duplication syndrome

MELAS

Menkes disease

Metachromatic leukodystrophy

Molybdenum cofactor deficiency

Mucolipidosis type I

Mucolipidosis type II

Mucolipidosis type IV

Multiple Sulfatase Deficiency

Neurodegeneration with brain iron accumulation OR NBIA (broad term)

Niemann-Pick type C

Pelizaeus-Merzbacher disease

PKAN OR NBIA

POLG-related disease

POLR3-related leukodystrophies

Pyruvate dehydrogenase deficiency

Rett syndrome

Sanfilippo syndrome OR Mucopolysaccharidosis 3 OR MPS3

Saposin A Deficiency

Saposin B Deficiency

Saposin C Deficiency

Schindler Disease

Sialic Acid Storage Disease

SLC5A6 Deficiency

Sly Syndrome OR Mucopolysaccharidosis Type 7 OR MPS7

Sulphite/Sulfite Oxidase Deficiency

Vanishing White Matter Disease

Wilson Disease

X-linked Adrenoleukodystrophy OR X-ALD

Zellweger spectrum OR infantile Refsum disease OR neonatal adrenoleukodystrophy OR Zellweger syndrome
