# Supplementary material for: Characterizing circulating biomarkers for childhood dementia disorders: A scoping review of clinical trials
Source: Neurotherapeutics. 2025 Feb 12;22(2):e00546. doi: 10.1016/j.neurot.2025.e00546 (PMC12014410; doi:10.1016/j.neurot.2025.e00546)
Supplement: Multimedia component 1 [file mmc1.docx]

**Supplementary Table 1:** Individual participant data sharing plans in childhood dementia clinical studies incorporating circulating biomarkers.

| **Study design** | **Records (n)** | **Individual participant data plan for sharing, n (% of records)** | **Individual participant data specified as withheld, n (% of records)** | **Individual participant data plan for sharing unknown or undecided, n (% of records)** |
| --- | --- | --- | --- | --- |
| **Observational** | 64 | 9 (14.1) | 44 (68.8) | 11 (17.2) |
| **Interventional** | 198 | 13 (6.6) | 143 (72.2) | 42 (21.2) |
| **Total (Either)** | 262 | 22 (8.4) | 187 (71.4) | 53 (20.2) |

**Supplementary Table 2:** The findings and contribution of biofluid disease-related biomarkers related to **primary pathophysiological pathway** in clinical studies of childhood dementia.

| **Biomarker**  **(Biofluid)** | **Condition** | **Intervention** | **Phase** | **Findings** | **Interpretation and Comments** | **Study** |
| --- | --- | --- | --- | --- | --- | --- |
| Cholesterol precursors: lanosterol, lathosterol, desmosterol  Cholesterol metabolites:4β-, 24S-, 25-, 27-hydroxycholesterol (s) | Niemann-Pick Type C | Small molecule:  IV Trappsol Cyclo  HP-β-CD | 1/2 | Decreased lathosterol, increased hydroxycholesterols. | Response/Pharmacodynamic | 2015-005761-23 |
|  |  |  |  | Reduced cholesterol precursors and increased metabolites, including 24S-hydroxycholesterol (CNS-specific). Trend towards normalization. Improved clinical outcomes. | Evidence of biological activity - interpreted as release of CNS-stored cholesterol and then reset of cholesterol homeostasis.  Response/Pharmacodynamic | [1]  NCT02912793 |
| *N*-palmitoyl-*O* phosphocholineserine (p) |  |  |  | Rapid reduction to below baseline. Improved clinical outcomes. |  |  |
| Unesterified Cholesterol (pbmc) | Niemann-Pick Type C | Small molecule: Arimoclomol | 2/3 | Increased from baseline for treated and placebo, but less for treated. | Measure of NPC lipid burden. Response/Pharmacodynamic | [2] NCT02612129 |
|  |  | Observational | -* | Elevated compared to HCs; increased over time. | NPC diagnosis, longitudinal monitoring  Monitoring potential Diagnostic. | [3]  NCT02435030 |
| Cholesterol esterification (pbmc) |  |  |  | Lower compared to HCs. No change over time or with clinical progression. No correlation with disease severity. | Biochemical diagnosis, independent of disease severity.  Monitoring, potential Diagnostic. |  |
| Cholestane-3β,5α,6β-triol (s) | Niemann-Pick type C | Observational | -* | Elevated compared to HCs. Increased over time. Positively correlated with greater disease severity on clinical scores. Not correlated with onset age. | Supports use as disease monitoring biomarker.  Monitoring | [3]  NCT02435030 |
|  |  | Small molecule: Arimoclomol | 2/3 | Non-significant decrease compared to placebo control. | Monitoring/Response, Diagnostic | [2] NCT02612129 |
| Lyso-sphingomyelin-509 (p) | Niemann-Pick type C | Small molecule: Arimoclomol | 2/3 | Elevated compared to HCs. Baseline correlated with NPCCSS score, serum cholestane-triol, and inversely with age of onset of neurological disease. *Post-treatment:* reduction compared to placebo. | Associated with disease severity. Evidence of biological activity, Regulatory approval not obtained, despite promise.  Monitoring/Response | [2]  NCT02612129 |
| 7-ketocholesterol (p) | Niemann-Pick type C | Lithium carbonate | 1* | *Post-treatment* decrease. Improved NPC Neurological Severity Scores. | Disease-specific biomarker linked to disease severity.  Response/Surrogate endpoint | [4]  NCT03201627 |
| Cholestane-3β, Bile acid B (5α), C-Triol (6β-triol) (p) | Niemann-Pick type C | Small molecule:  IV and IT VTS-270 (HP-β-CD) | 1/2* | Reduced with low dose. | NPC1-specific pharmacodynamic biomarkers. Response/Pharmacodynamic | NCT03887533 |
| Cholestane-3β,5α,6β-triol and 7-ketocholesterol (p) | Niemann-Pick type C | Small molecule: NAC | 1/2* | *Post-treatment* no significant change, oxidate stress not reduced. | Response/Surrogate endpoint | [5] NCT00975689 |
|  |  | Observational | - | Elevated compared to HCs. Higher levels correlated with earlier disease onset, greater severity. | Disease course monitoring alone or as numerator of oxysterol index ratio.  Monitoring | [6]  06-CH-0186 / NCT00344331 |
| 24(S)-hydroxycholesterol (p) |  |  |  | Decreased compared to HCs and inversely correlated with disease severity. | Denominator in oxysterol index for disease monitoring.  Monitoring |  |
| Cholestane-3β,5α,6β-triol (CSF) |  |  |  | Elevated compared to HCs, along with precursor 5β,6β-epoxycholesterol | Part of disease’s distinct oxysterol profile  Monitoring |  |
| 7α,27 & 7β,27 hydroxy cholesterol (CSF) |  |  |  | Both reduced compared to HCs. |  |  |
| 24(S)-hydroxycholesterol (CSF) | Niemann-Pick type C | Small molecule:  IT HP-β-CD | 1* | *Post-treatment*: Significant increase | Pharmacodynamic evidence of improved cholesterol metabolism. With FABP3 and calbindin, supports clinical development and phase 2/3.  Response/Pharmacodynamic | (Ory, Ottinger et al. 2017)  NCT01747135 |
| 24(S)-hydroxycholesterol (p) |  |  |  | *Post-treatment*: Increased. |  |  |
| Fatty acid binding protein 3, FABP3 (CSF) |  |  | 1 | Baseline: Higher compared to HCs *Post-treatment*: Decrease from baseline | Pharmacodynamic evidence of shift in CNS pathology towards normal.  Response/Pharmacodynamic |  |
| Calbindin 1/D (CSF) |  |  |  |  |  |  |
| Calretinin, CALB2 (CSF) |  | Observational | - | Elevated compared to controls. Positively correlated with clinical scores and neurological onset age. | Potential as prognostic biomarker.  Monitoring, potential Prognostic | [7] NCT00344331 |
| Sulfatide (CSF) | MLD | IV ERT:  rh ASA  (Metazym) | 1/2* | Baseline: >20x HCs, correlation with higher albumin index. *Post-treatment*: decrease relative to baseline. No clinical efficacy. | Needs more research to judge as disease severity marker. Showed ERT did not cross blood-brain barrier sufficiently, but some PNS benefits. Suggest future clinical development via IT route.  Response/Pharmacodynamic | [8]  NCT00418561 / 2006-005341-11 |
|  |  | IT ERT:  rh ASA | 1/2 | Decrease in CSF sulfatide and lysosulfatide to within normal range. | Despite general clinical decline, biomarker changes support further development.  Response/Pharmacodynamic | [9]  NCT01510028 |
|  |  | ERT: rhASA (HGT-1111) | 2 | Impact unclear, possibly stable. | Response/Pharmacodynamic, Monitoring. | 2008-000084-41 |
|  |  | IV ERT: rhASA (Metazym) | 2 | Decreased with higher doses. | Response/Pharmacodynamic | 2007-006345-40 |
| Sulfatide (u) |  | IV ERT:  rh ASA  (Metazym) | 1/2 | Inclusion criteria: MLD diagnosis based partly on elevated urinary sulfatide. | Diagnostic | [8]  NCT00418561 / 2006-005341-11 |
|  |  | IT ERT:  rh ASA | 1/2 |  |  | [9]  NCT01510028 |
| Arylsulfatase A activity (CSF, HSC, PBMC) | MLD | Cell and Gene Therapy:  LV-HSC-GT  *ARSA* | 1/2* | Post-treatment, sustained increase in ARSA activity with clinical benefit | Seeking regulatory approval.  Response/Surrogate Endpoint | [10-12]  NCT01560182 |
| Disease-specific enzyme levels | MLD  Krabbe  X-ALD  MPS I/II | HSC transplant with reduced-intensity conditioning | 2 | Descriptive narrative of mixed cohort with change in enzyme activities to the unaffected range and varied clinical improvements. | Response/Surrogate Endpoint | [13]  NCT01962415 |
| α-N-acetylglucosaminidase activity (CSF) | MPS III  Sanfilippo | IC Gene:  AAV-NAGLU cDNA | 1/2 | Improved from undetectable to detectable. Improved neurocognitive progression. | Potential surrogate marker of clinical efficacy  Response/Surrogate Endpoint | [14]  NCT03300453 / 2012-000856-33 |
| Glycosaminoglycans including:  Heparan sulfate  Dermatan sulfate (CSF) | MPS II  (Hunter) | Observational | -* | Elevated levels link to cognitive impairment. Attenuated phenotype exceeded non-MPS but overlap with cognitively affected MPS subjects. | Possible diagnostic or prognostic tool to identify people at risk of developing cognitive impairment.  Monitoring, potential Diagnostic or Prognostic | [15]  NCT01449240 |
|  |  | IT ERT:  rh iduronate-2-sulfatase (I2S) (Idursulfase) | 1/2 | Post-treatment significantly reduced. | Supports further development.  Response/Pharmacodynamic | [16] NCT00920647 |
|  | MPS III  (Sanfilippo) | Observational | - | Elevated compared to HCs; relatively constant over time. | No relationship with disease progression.  Monitoring | [17] NCT01047306 |
|  |  | IT ERT:  rh heparan-n-sulfatase (HNS) | 1/2 | Non-dose-dependent decrease. Did not slow neurocognitive decline. | Pre-specified efficacy criteria not met, terminated early.  Response/Pharmacodynamic | [18]  NCT01299727 |
|  | MPS II (Hunter)  MPS III  (Sanfilippo) |  | 2b | Reduced, but not significantly different to controls. Coincided with metabolic improvements. | Primary neurocognitive efficacy endpoint not met.  Response/Pharmacodynamic | [19]  NCT02060526 |
|  | MPS III  (Sanfilippo) | IV ERT:  CM - rh sulfamidase | 1/2 | Reduced level. Clinical benefit: stabilised neurocognitive assessment scores. | Response/Pharmacodynamic | [20] NCT03423186 and NCT03811028 |
|  | MPS III  (Sanfilippo) | IT ERT:  rh heparan-n-sulfatase (HNS) | 1/2 | Baseline elevated; post-treatment declined; 6/12 subjects neurodevelopmentally stable. | Response/Pharmacodynamic | [21]  NCT01155778 |
|  | MPS III  (Sanfilippo) | Gene:  AAV-hSGSH | 1/2* | Decreased after treatment. | Response/Pharmacodynamic | NCT04088734 |
|  | MPS III  (Sanfilippo) | Small molecule:  Genistein aglycone | 3 | Non-significantly reduced from placebo, no clinical benefit. | Response/Pharmacodynamic | 2013-001479-18 |
|  | MPS III  (Sanfilippo | IT ERT:  rh.HNS | 2 | Impact unclear. | Response/Pharmacodynamic | NCT02350816 |
|  | MPS I  (Hurler) | IV ERT:  (Valanafusp alfa) HIRMAb-IDUA | 1/2 | No significant change. Coincided with stabilized CNS and somatic function. | Response/Pharmacodynamic | [22] NCT03053089 and NCT03071341 |
|  | MPS I  (Hurler) | Cell and Gene Therapy:  LV-HSPC-GT  *IDUA* | 1/2 | Localized clearance. Cognitive measures stabilized, function measures stable or improved. | Response/Pharmacodynamic | [23]  NCT03488394 |
| Heparan sulfate and nonreducing ends (CSF, p) | MPS III  (Sanfilippo) | Observational | -* | Elevated compared to HCs, stable over time. No correlation with development quotient, or cortical gray matter volume. | Not suited as biomarkers of disease severity or progression. Accumulation indicative of disease process. Normalization acceptable as therapeutic goal.  Monitoring | [24]  NCT02493998 |
| Heparan sulfate (p) | MPS III  (Sanfilippo) | Gene:  AAV-hSGSH | 1/2 | Decreased with treatment. | Response/Pharmacodynamic | NCT04088734 |
|  |  | Small molecule:  Genistein aglycone | 3 | Not meaningfully different from placebo. | Response/Pharmacodynamic | 2013-001479-18 |
| Glycosaminoglycans including:  Heparan sulfate  Dermatan sulfate  Chondroitin sulfate (u) | MPS II  (Hunter) | IT ERT:  rh iduronate-2-sulfatase (I2S) (Idursulfase) | 1/2 | No apparent effect. | Response/Pharmacodynamic | [16] NCT00920647 |
|  | MPS III  (Sanfilippo) | Observational | - | Elevated compared to controls; relatively constant over time. | No relationship with disease progression.  Monitoring | [17] NCT01047306 |
|  | MPS II (Hunter)  MPS III  (Sanfilippo) | IT ERT:  rh heparan-n-sulfatase (HNS) | 2b | Reduced post-treatment. Coincided with metabolic improvements. | Primary neurocognitive efficacy endpoint not met.  Response/Pharmacodynamic | [19]  NCT02060526 |
|  | MPS III  (Sanfilippo) | IV ERT:  CM rh sulfamidase | 1/2 | Reduced level. Clinical benefit: stabilised neurocognitive assessment scores. | Response/Pharmacodynamic | [20] NCT03423186 and NCT03811028 |
|  | MPS III  (Sanfilippo) | IT ERT:  rh heparan-n-sulfatase (HNS) | 1/2 | Baseline elevated; post-treatment declined; 6/12 subjects neurodevelopmentally stable. | Response/Pharmacodynamic | [21]  NCT01155778 |
|  | MPS III  (Sanfilippo) | Gene:  AAV-hSGSH | 1/2 | Decreased with treatment. | Response/Pharmacodynamic | NCT04088734 |
|  | MPS III  (Sanfilippo) | Small molecule:  Genistein aglycone | 3 | Not meaningfully different from placebo. | Response/Pharmacodynamic | 2013-001479-18 |
|  | MPS III  (Sanfilippo | IT ERT:  rh.HNS | 2 | Impact unclear. | Response/Pharmacodynamic | NCT02350816 |
|  | MPS I  (Hurler) | Gene:  AAV-ZFN-IDUA | 1/2 | Showed no correlation with transient increase in leukocyte enzyme activity. | No long-term enzyme expression  Response/Pharmacodynamic | [25]  NCT02702115 |
|  | MPS I  (Hurler) | IV ERT:  IDUA  (Laronidase) | 1/2 | Reduced, but not to normal range. | Testing for target engagement.  Response/Pharmacodynamic | [26]  NCT00741338 |
|  |  |  | 2 | Decreased with treatment. | Response/Pharmacodynamic. | NCT00146757 |
|  |  |  | 3 | Decreased, with improved clinical scores. | Response/Pharmacodynamic. | [27] NCT00146770 |
|  |  |  | 3 | Decreased with treatment. | Response/Pharmacodynamic. | NCT00258011 |
|  |  |  | 4 | Decreased with treatment. | Response/Pharmacodynamic. | NCT00144781 |
|  | MPS I  (Hurler) | IV ERT:  (Valanafusp alfa) HIRMAb-IDUA | 1/2 | Level stable with treatment. Coincided with stabilized CNS and somatic function. | Response/Pharmacodynamic | [22]  NCT03053089 and NCT03071341 |
|  | MPS I  (Hurler) | Cell and Gene Therapy:  LV-HSPC-GT  *IDUA* | 1/2 | Decreased to normal levels; Clinical improvement or stability. | Response/Pharmacodynamic | [23]  NCT03488394 |
|  | MPS VII  (Sly) | IV ERT: rhGUS (vestronidase alfa) | 1/2 | Decreased >50% from baseline. | Response/Pharmacodynamic | 2013-001152-35 |
|  |  |  | 3 | Decreased from baseline. | Response/Pharmacodynamic | 2014-005638-71 |
|  |  | Observational | - | Untreated, elevated. Treated (vestronidase alfa), decreased. | Monitoring, Response/Pharmacodynamic | [28] NCT03604835 |
| Heparan sulfate (s) | MPS III  (Sanfilippo) | IV ERT:  CM rh sulfamidase | 1/2 | Reduced level. Clinical benefit: stabilised neurocognitive assessment scores. | Response/Pharmacodynamic | [20] NCT03423186 and NCT03811028 |
| α-L-iduronidase activity (p, leukocyte) | MPS I  (Hurler) | Gene:  AAV-ZFN-IDUA | 1/2 | No detectable enzyme activity, despite transient increase in leukocyte enzyme activity. | No lasting enzyme expression.  Response/Pharmacodynamic | [25]  NCT02702115 |
| α-L-iduronidase activity (CSF) | MPS I  (Hurler) | Cell and Gene Therapy:  LV-HSPC-GT  *IDUA* | 1/2* | Improved to reach detectable levels; associated with local GAG clearance; cognitive measures stabilized, function measures stable or improved. | Response/Pharmacodynamic | [23]  NCT03488394 |
| Granular Osmophilic Deposits (leukocyte) | NCL  (Battens Disease) | Small molecule:  Cysteamine bitartrate (Cystagon)  N-acetylcysteine | 4* | Less per cell and fewer cells with deposits Progressive brain atrophy continued. Reduced irritability. | Suggest less ceroid accumulation.  Response/Pharmacodynamic | [29]  NCT00028262 |
| VLCFA  C26:C22 ratio (p) | Zellweger | Small molecule:  Betaine | 3* | Large intra- and inter-individual variations in peroxisome functions.  Elevated ratio used for biochemical diagnosis and inclusion criteria. | Blood metabolite markers may be less sensitive than direct measurements of cell responses.  Response/Pharmacodynamic, Diagnostic. | NCT01838941 |
| VLCFA  C26:0 (blood) | X-ALD | Dietary Supplement:  GTO/GTE | 2 | Limited detail, baseline mean only. Elevation as inclusion criteria. | Response/Pharmacodynamic, Diagnostic. | NCT00004418 |
| Global DNA methylation (blood) | Rett syndrome | Dietary Supplement:  Creatine monohydrate | -* | Increased global DNA methylation. | Response/Pharmacodynamic | [30] NCT01147575 |
| Methionine, Homocysteine, S-adenosylmethionine, S-adenosylhomocysteine  (blood) | Rett syndrome | Dietary Supplement:  Creatine monohydrate | - | Metabolic markers of DNA methylation cycle were unchanged. | Response/Pharmacodynamic | [30]  NCT01147575 |
| Sialotransferrin isoforms (s) | PMM2-Congenital disorder of glycosylation | Dietary Supplement:  D-Galactose | - | Glycosylation profile not improved. Positive trend in monosialo transferrin/disialotransferrin ratio. No correlation with clinical improvement. | Recommends larger placebo-controlled study to judge benefit in milder patients.  Response/Pharmacodynamic | [31]  NCT02955264 |
| Arginine, ornithine, glutamate, lactate, and citrulline (s) | MELAS | Dietary Supplement:  L-arginine | 2 | Baseline: Lower arginine, no difference ornithine, citrulline than HCs. *Post-treatment*: Increased arginine, ornithine. No change citrulline, lactate, glutamate. Improved cerebrovascular reactivity. | Efficacy evidence; suggested improved blood flow.  Response/Pharmacodynamic | [32, 33]  NCT01603446 |
| Lactate (blood) | MELAS | Small molecule: Idebenone | 2 | High dose increased, low dose decreased, placebo decreased more. | Response/Pharmacodynamic | NCT00887562 |
| Mannose-rich oligosaccharides (s) | Alpha-Mannosidosis | IV ERT:  rh. Lysosomal α -mannosidase | 2 | Decrease from baseline. Clinical improvement | Response/Surrogate Endpoint. | [34]  NCT02998879 |
|  |  | IV ERT:  rh.alpha-mannosidase (velmanase alfa) | 1/2* & 3* | Decrease from baseline. Clinical improvement (motor, cognitive.) | Co-primary endpoint. Reduced oligosaccharide accumulation.  Response/Surrogate Endpoint | [35] NCT01681953 / 2012-000979-17 and NCT01908725 / 2013-000321-31 [36] NCT01285700 / 2010-022085-26 |
|  |  |  | 3* | Significant clearance. Clinical improvement | Efficacy marker.  Response/Surrogate Endpoint | [37]  NCT01908712 / 2013-000336-97 |
| Mannose-rich oligosaccharides (CSF, urine) | Alpha-Mannosidosis | IV ERT:  rh.alpha-mannosidase (velmanase alfa) | 1/2* | Decrease from baseline. Clinical improvement (motor and cognitive) | Efficacy marker.  Response/Surrogate Endpoint | [36]  NCT01285700 / 2010-022085-26 |
|  |  |  | 3 | Decrease from baseline | Response/Pharmacodynamic | [37]  NCT01908712 / 2013-000336-97 |
| Mannose-rich oligosaccharides  (CSF) | Alpha-Mannosidosis | Observational | - | Higher CSF oligosaccharides correlated with lower equivalent age in memory screening test. | Genotype-phenotype correlation depends on the subcellular localisation of mutant MAN2B1 protein to the lysosome.  Monitoring, potential Prognostic. | [38, 39]  NCT01681953 / 2012-000979-17 |
| S-sulfocysteine (u) | Molybdenum Cofactor Deficiency | Small molecule: Fosdenopterin (ORGN001) | 2 | Decreased from baseline post-treatment. Clinical appears stable. | Clinical appears stable.  Response/Pharmacodynamic | NCT02047461 / 2013-002701-56 |
| Lyso-sphingomyelin (p) | Acid Sphingo-myelinase Deficiency | IV ERT:  rh. acid sphingo-myelinase  (olipudase alfa) | 1/2 | Elevated at baseline, stable decrease post-treatment, nearing ULN. No change in neurological examination. | Response/Pharmacodynamic | [40, 41] NCT02292654, NCT02004704 / 2013-000051-40 |
| Sphingomyelin (p) |  |  |  | Decreased post-treatment. | Response/Pharmacodynamic |  |
| Lipid profile: total cholesterol, LDL-C, HDLC-C and TG (p) |  |  |  | Baseline elevated total cholesterol, LDL-C, TG, below normal HDL-C; all normalised post-treatment. | Response/Pharmacodynamic |  |
| *Results were reported for 51/111 studies identified as being completed at censoring.* | | | | | | |

**ABBREVIATIONS:** ***** studies in which candidate biomarker was a primary endpoint. **3MSCT:** Three Minute Stair Climb Test. **AAV**: adeno-associated viral vector. **ASA:** Arylsulfatase A. **CDG**: Congenital disorder of glycosylation**. CM:** chemically modified. **CNS**: Central nervous system. **CSF:** cerebrospinal fluid. **ERT:** Enzyme replacement therapy. **FABP3:** Fatty Acid Binding Protein 3. **GT:** Gene Therapy. **GTO/GTE:** Glyceryl trierucate/glyceryl trioleate (Lorenzo’s oil). **GUS:** Beta-Glucuronidase. **h/H:** Human. **HC:** Health control. **HDL-C**: High density lipid cholesterol. **HIRMAb**: Human insulin receptor monoclonal antibody. **HPBCD/HP-β-CD**: hydroxypropyl-β-cyclodextrin. **HS(P)C**: Hemopoietic Stem (and Progenitor) Cells. **IC:** Intracerebral. **IDUA:** α-L-Iduronidase. **IT:** Intrathecal. **IV:** Intravenous. **LDL-C**: Low density lipid cholesterol. **LV:** Lentiviral vector. **MELAS**: Mitochondrial Encephalopathy, Lactic Acidosis, and Stroke-like episodes**. MLD:** metachromatic leukodystrophy. **MPS**: Mucopolysaccharidosis. **NAC:** N-acetylycysteine. **NCL:** Neuronal Ceroid Lipofuscinosis**. NPC:** Niemann-Pick Disease Type C. **p:** Plasma. **pbmc**: Peripheral Blood Mononuclear Cells. **PMM2-CDG**: Phosphomannomutase 2-congenital disorder of glycosylation. **PNS:** Peripheral nervous system. **rh:** recombinant human. **s:** Serum. **SGSH**: N-sulphoglucosamine sulphohydrolase. **TG:** triglycerides. **ULN**: Upper limit of normal. **VLCFA:** Very Long Chain Fatty Acid. **X-ALD**: X-linked adrenoleukodystrophy. **ZFN**: Zinc-finger nuclease.

**Supplementary Table 3:** The findings and contribution of circulating disease-related biomarkers associated with **downstream pathological processes** in clinical studies of childhood dementia

| **Biofluid and Biomarker** | **Condition** | **Intervention** | **Phase** | **Findings** | **Interpretation and Comments** | **Study** |
| --- | --- | --- | --- | --- | --- | --- |
| Cytoskeletal  Biomarker Panel  CSF  Tau  CSF  Glial Fibrillary Acidic Protein (GFAP)  CSF  Neurofilament Light Chain (NfL, NEFL) | Alpha-mannosidosis | IV ERT Lamazyn, velamanase alfa:  rh alpha-mannosidase | 1/2 | Tau: Elevated baseline.  Tau, GFAP: Post-treatment decrease.  Nfl: Non-statistically significant post-treatment decrease.  Associated with improved motor and cognitive function. | CSF biomarkers may indicate drug crosses blood-brain barrier.  Response/Pharmacodynamic | [36]  NCT01285700 / 2010-022085-26 |
|  |  |  | 3 | No change vs placebo control. Improved pediatric motor and pulmonary function. | Evidence of lack of biological activity in CNS  Response/Pharmacodynamic | [35]  NCT01681953 / 2012-000979-17 |
|  |  |  |  | No correlation with genotype or subcellular localization subgroups | Diagnostic/Stratification? | [38]  NCT01681953 / 2012-000979-17 |
|  |  |  |  | Elevated, negatively correlated with cognitive function compared to HCs | Early CNS neuropathology changes with gliosis and reduced myelination, relevant to planning treatment initiation  Prognostic (exploratory) | [42]  NCT01681953 / 2012-000979-17 |
|  |  |  | 1/2/3 | No significant change from baseline to last observation. | Lack of effect on CSF biomarkers is an unmet need and a future research focus.  Response/Pharmacodynamic | [37]  Data: NCT01285700 / 2010-022085-26, NCT01908725, NCT02478840 |
|  | Metachromatic Leukodystrophy MLD | IV ERT  Metazym:  rh ASA | 1/2 | Decreased from baseline; coincided with clinical outcome decrease, but association not reported. | Efficacy – Response/Pharmacodynamic | [8]  NCT00418561 / 2006-005341-11 |
| CSF  Total Tau | MPS III A (Sanfilippo) | Observational | - | Increase relative to HCs and decreased with increasing age. | No relationship with disease progression  Prognostic - Monitoring (secondary) | [17]  NCT01047306 |
| CSF  Phospho-Tau | MPS III A (Sanfilippo) | Observational | - | Increase relative to HCs and decreased with increasing age. | No relationship with disease progression  Prognostic - Monitoring (secondary) | [17]  NCT01047306 |
| CSF  Total Tau | Niemann-Pick Type C1  (NP-C1) | Small molecule:  IV Trappsol Cyclo  Hydroxypropyl- β -cyclodextrin  (HP-β-CD) | 1/2 | Baseline: Elevated compared to HCs in literature.  Post-treatment: Reduction trend, coincides with clinical improvement. | Evidence of drug biological activity and target engagement. Baseline elevated in NPC patients due to neurodegeneration. Reduction suggests rate of neuronal apoptosis decreased.  Efficacy – Response/Pharmacodynamic | [1]  NCT02912793 |
| CSF  Microtubule Associated Protein Tau  (MAPT) | Niemann-Pick type C  (NP-C) | Observational | - | Elevated compared to HCs. | Marker of neuronal damage.  Monitoring (exploratory)? | [7]  NCT00344331 |
| CSF  Neurofilament Light Chain (NfL, NEFL) | Rett syndrome | Small molecule: Fingolimod | 1/2 | CSF NfL = no correlation with clinical scores.  CSF NfL = smaller brain deep gray matter volume; lower perfusion. | Monitoring – Response/Pharmacodynamic (exploratory) | [43]  NCT02061137 |
|  | Niemann-Pick type C  (NP-C) | Observational  Oral Small Molecule:  Migulstat  IT Small Molecule:  Adrabetadex HPβCD | 1/2/3 | Baseline: Elevated compared to HCs.  Positively associated with symptom severity and clinical measures.  Post miglustat treatment: Decreased.  Post- HPβCD treatment: No change, may increase if adjusted for miglustat effect. | Potential utility as a biomarker in future therapeutic trials. General marker of neuronal damage – non-specific to NP-C-impacted cerebellar Purkinje neurons.  Monitoring (disease progress)  Efficacy – Response/Pharmacodynamic | [44]  NCT00001367, NCT01747135, NCT02534844, IND 119856 |
|  |  | Observational | - | Elevated compared to HCs. | Concurs with plasma NfL elevation.; known neuronal damage marker.  Monitoring (exploratory) | [7]  NCT00344331 |
| SERUM  Neurofilament Light Chain (NfL, NEFL) | Rett syndrome | Small molecule: Fingolimod | 1/2 | Baseline: No difference to HCs.  No change over time, before or during treatment.  No correlation with clinical scores.  Higher linked to lower brain perfusion. | Evidence against Rett as neurodegenerative.  Efficacy – Response/Pharmacodynamic (exploratory) | [43]  NCT02061137 |
| CSF  Calbindin 1/D  (CALB1) | Niemann-Pick type C  (NP-C) | Observational | - | Elevated compared to HCs. | Neuronal damage marker, especially cerebellar Purkinje neurons – loss linked to NP-C1 cerebellar ataxia.  Monitoring (exploratory) | [7]  NCT00344331 |
| CSF  Brain Derived Neurotrophic Factor (BDNF) | Rett syndrome | Small molecule: Fingolimod | 1/2* | No change in level over time.  Associated with all clinical scores.  Higher BDNF better score.  No correlation with serum BDNF. | Potential biomarker for Rett syndrome.  Higher level linked with better clinical outcome scores.  Treatment: No observed effect on CSF BDNF or on neurophysiological and clinical outcomes.  Monitoring, Efficacy – Response/Pharmacodynamic | [43]  NCT02061137 |
| SERUM  Brain Derived Neurotrophic Factor (BDNF) | Rett syndrome | Small molecule: Fingolimod | 1/2* | Baseline: No difference compared to HCs.  No change in levels over time, before or during treatment.  No correlation with clinical scores.  Associated with brain deep gray matter volumes.  No correlation with CSF BDNF. | Need further evaluation as biomarker for Rett syndrome.  Treatment: No observed effect on serum BDNF or on neurophysiological and clinical outcomes.  Monitoring, Efficacy – Response/Pharmacodynamic | [43]  NCT02061137 |
|  |  | Other:  Enriched environment | -* | Baseline lower than general population. Increased from baseline, alongside gains in gross motor skills. | Monitoring, Efficacy - Response | [45]  ACTRN12615001286538 |
| Global DNA methylation (blood) | Rett syndrome | Dietary Supplement:  Creatine monohydrate | -* | Increased global DNA methylation. | Response/Pharmacodynamic | [30] NCT01147575 |
| WHOLE BLOOD  Glutathione | X-ALD | Diet supplement:  Vitamin D | 1 | No change in blood glutathione | MRS shows increased brain glutathione. Warrants further study.  Efficacy – Response/Pharmacodynamic | [46]  NCT02595489 |
| Peripheral Blood Mononuclear Cells  PBMC  Heat Shock Protein 70 (HSP70) | Niemann-Pick type C | Observational | -* | Baseline: Lower than HCs. Stable over time. No correlation with disease severity. | Relevance: HSP70 ensures proper NPC1 protein function.  NPC treatment drug ‘Arimoclomol’ functions as HSP70 amplifier.  Monitoring | [3]  NCT02435030 |
|  |  | Small molecule: Arimoclomal  (HSP70 inducer) | 2/3 | Increased in response to 12 months treatment. | Functioned as drug activity pharmacodynamic marker. Evidence for target engagement and mechanism of action via Heat Shock Response activation.  Efficacy - Response/Pharmacodynamic | [2]  NCT02612129 |
| CSF  Chitotriosidase-1 (CHIT1) | Niemann-Pick type C | Observational | - | Elevated vs control | Matches prior plasma findings [47].  Monitoring (exploratory) | [7]  NCT00344331 |
|  | Metachromatic Leukodystrophy MLD | IV ERT  Metazym:  rh ASA | 1/2 | Post-treatment: Change relative to baseline, inconclusive: some increase, some reduce. | No information about relation to clinical outcomes.  Response/Pharmacodynamic | [8]  NCT00418561 / 2006-005341-11 |
| CSF, SERUM  Albumin | MLD | IV ERT:  rh ASA  (Metazym) | 1/2 | CSF/serum albumin index high, correlated with higher CSF sulfatides. *Post-treatment*: decreased from baseline. | Indicates impaired blood-brain barrier integrity. Potential marker of disease severity, but variable accuracy.  Response/Pharmacodynamic, potential Prognostic. | [8]  NCT00418561 / 2006-005341-11 |
| PLASMA  Chitotriosidase-1 (CHIT1) | Acid Sphingo-myelinase Deficiency | IV ERT:  rh. acid sphingo-myelinase  (olipudase alfa) | 1/2 | Elevated at baseline, stable decrease post-treatment, near ULN 24-months. No change in neurological examination. | Response/Pharmacodynamic | [40, 41] NCT02292654, NCT02004704 / 2013-000051-40 |
| BLOOD  Chemokine CCL18 |  |  |  | Decreased post-treatment. | Response/Pharmacodynamic |  |
|  | Gaucher Disease Type 3 | IV ERT:  Velaglucerase alfa: β-gluco-cerebrosidase | 1/2 | From elevated baseline, decreased over time. No positive impact on neurological abnormalities (5/6 participants). | Efficacy marker.  Diagnostic  Response/Pharmacodynamic | [48]  NCT01685216. |
| BLOOD  Chitotriosidase-1 (CHIT1) |  |  |  |  |  |  |
| CSF  Chemokine CCL18 | Niemann-Pick type C | Observational | - | Elevated vs control. Correlate with age of neurological onset and phenotypic aspects. | Potential as prognostic.  Monitoring, Prognostic?? (exploratory) | [7]  NCT00344331 |
| CSF  Cytokines:  IL-1β, IL-1Ra, IL-2, IL-4, IL-5, IL-6, IL-7, IL-9, IL-10, IL-12-p70, IL-13, IL-15, IL-17, IFN-γ, TNF-α | MPS IIIB  (Sanfilippo) | Gene therapy:  Intra-parenchymal  rAAV2/5 human α -N-acetylglucosaminidase (NAGLU)  with immuno-suppression | 1/2* | Baseline: Elevated IL-9, IL-15, IL-13, IFN-γ, and IL-7 compared to healthy children in literature.  Post-treatment: Unchanged/stable. One patient increased IFN-γ and IL-1Ra, two patients fluctuating IL-7. | Baseline: neuroinflammation and peripheral inflammation.  Treatment did not trigger neuroinflammation.  Recommends systematic standardized immuno-monitoring approach for safety, efficacy, inflammatory management, and biomarker identification.  Response/Pharmacodynamic | [49]  NCT03300453 / 2012-000856-33 |
| CSF Chemokines and growth factors: IL-8, IP-10, Eotaxin, basic FGF, G-CS F, GM-CS F, MCP1, MIP-1α, MIP-1β, PDGF, RANTES, VEGF |  |  |  | Baseline: Elevated GM-CS F and G-CS F compared to healthy children in literature.  Post-treatment: Unchanged/stable. IP-10 increase/decrease different patients. |  |  |
| PLASMA  Cytokines:  IL-1β, IL-1Ra, IL-2, IL-4, IL-5, IL-6, IL-7, IL-9, IL-10, IL-12-p70, IL-13, IL-15, IL-17, IFN-γ, TNF-α |  |  |  | Baseline: Elevated IL-7, IL-9, TNF-α, IL-10, IL-6, IFN-γ, and IL-1Ra compared to healthy children in literature.  Post-treatment: Unchanged/stable, one patient increased IL-1Ra. |  |  |
| PLASMA  Chemokines and growth factors: IL-8, IP-10, Eotaxin, basic FGF, G-CSF, GM-CSF, MCP1, MIP-1α, MIP-1β, PDGF, RANTES, VEGF |  |  |  | Baseline: Elevated IL-8, G-CSF, MIP-1β, GM-CSF, IP-10, and RANTES compared to healthy children in literature.  Post-treatment: Unchanged/stable. |  |  |
| CSF  TNF-α | Niemann-Pick Type C1 | Small molecule:  IV Trappsol Cyclo  (HP-β-CD) | 1/2 | No information gained, below level of method quantification | CNS inflammation marker.  Response/Pharmacodynamic | [1]  NCT02912793 |
| CSF  GFAP |  |  |  | No information, below level of method quantification. | CNS inflammation marker.  Response/Pharmacodynamic | [1]  NCT02912793 |
| *Results were reported for 15/40 studies identified as being completed at censoring, including integrated analyses of several studies.* | | | | | | |

**ABBREVIATIONS:** ***** candidate biomarker was a primary endpoint. **AAV**: adeno-associated viral vector. **ASA:** Arylsulfatase A. **b**. blood. **BDNF:** Brain Derived Neurotrophic Factor. **CHIT1**: Chitotriosidase-1. **CNS**: Central nervous system**. CSF:** cerebrospinal fluid**. DNA:** Deoxyribonucleic Acid. **ERT:** Enzyme replacement therapy. **FGF:** fibroblast growth factor. **G-CS F:** Granulocyte colony-stimulating factor.  **GFAP**: Glial Fibrillary Acidic Protein. **GM-CS F**: Granulocyte-macrophage colony-stimulating factor. **HC:** Healthy Control. **HPBCD/HP-β-CD**: hydroxypropyl-β-cyclodextrin. **HSP70**: Heat Shock Protein 70. **IC:** Intracerebral. **IFN**: Interferon. **IL**: Interleukin. **IP-10**: Interferon gamma-induced protein 10. **IT:** Intrathecal. **IV:** Intravenous. **MAPT**: Microtubule Associated Protein Tau. **MCP-1:** monocyte chemoattractant protein-1. **MIP:** Macrophage Inflammatory Protein. **MLD:** metachromatic leukodystrophy. **MPS**: Mucopolysaccharidosis. **MRS:** magnetic resonance spectroscopy. **NAGLU:** α-N-acetylglucosaminidase. **NEFL/NfL** Neurofilament. **NPC/NP-C:** Niemann-Pick Disease Type C. **p:** Plasma. **pbmc**: Peripheral Blood Mononuclear Cells. **PD:** Pharmacodynamic. **PDGF**: Platelet-derived growth factor. **RANTES**: Regulated on Activation, Normal T-cell Expressed and Secreted**. rh:** recombinant human. **s:** Serum. **TNF-α**: Tumour Necrosis Factor Alpha. **ULN**: Upper limit of normal. **VEGF**: Vascular endothelial growth factor. **wb:** whole blood. **X-ALD**: X-linked adrenoleukodystrophy.

Bibliography – Supplementary Tables

1. Sharma, R., et al., *Long-term administration of intravenous Trappsol® Cyclo™ (HP-β-CD) results in clinical benefits and stabilization or slowing of disease progression in patients with Niemann-Pick disease type C1: Results of an international 48-week Phase I/II trial.* Molecular Genetics and Metabolism Reports, 2023. **36**.

2. Mengel, E., et al., *Efficacy and safety of arimoclomol in Niemann‐Pick disease type C: Results from a double‐blind, randomised, placebo‐controlled, multinational phase 2/3 trial of a novel treatment.* Journal of Inherited Metabolic Disease, 2021. **44**(6): p. 1463-1480.

3. Mengel, E., et al., *Clinical disease progression and biomarkers in Niemann–Pick disease type C: a prospective cohort study.* Orphanet Journal of Rare Diseases, 2020. **15**(1).

4. Han, S., et al., *Potential Disease-Modifying Effects of Lithium Carbonate in Niemann-Pick Disease, Type C1.* Frontiers in Pharmacology, 2021. **12**.

5. Fu, R., et al., *Efficacy of N-acetylcysteine in phenotypic suppression of mouse models of Niemann–Pick disease, type C1.* Human Molecular Genetics, 2013. **22**(17): p. 3508-3523.

6. Porter, F.D., et al., *Cholesterol Oxidation Products Are Sensitive and Specific Blood-Based Biomarkers for Niemann-Pick C1 Disease.* Science Translational Medicine, 2010. **2**(56): p. 56ra81-56ra81.

7. Campbell, K., et al., *Identification of cerebral spinal fluid protein biomarkers in Niemann-Pick disease, type C1.* Biomarker Research, 2023. **11**(1).

8. Í Dali, C., et al., *Intravenous arylsulfatase A in metachromatic leukodystrophy: a phase 1/2 study.* Annals of Clinical and Translational Neurology, 2021. **8**(1): p. 66-80.

9. í Dali, C., et al., *Safety of intrathecal delivery of recombinant human arylsulfatase A in children with metachromatic leukodystrophy: Results from a phase 1/2 clinical trial.* Molecular Genetics and Metabolism, 2020. **131**(1): p. 235-244.

10. Biffi, A., et al., *Lentiviral Hematopoietic Stem Cell Gene Therapy Benefits Metachromatic Leukodystrophy.* Science, 2013. **341**(6148): p. 1233158.

11. Sessa, M., et al., *Lentiviral haemopoietic stem-cell gene therapy in early-onset metachromatic leukodystrophy: an ad-hoc analysis of a non-randomised, open-label, phase 1/2 trial.* The Lancet, 2016. **388**(10043): p. 476-487.

12. Fumagalli, F., et al., *Lentiviral haematopoietic stem-cell gene therapy for early-onset metachromatic leukodystrophy: long-term results from a non-randomised, open-label, phase 1/2 trial and expanded access.* The Lancet, 2022. **399**(10322): p. 372-383.

13. Vander Lugt, M.T., et al., *Reduced-intensity single-unit unrelated cord blood transplant with optional immune boost for nonmalignant disorders.* Blood Advances, 2020. **4**(13): p. 3041-3052.

14. Tardieu, M., et al., *Intracerebral gene therapy in children with mucopolysaccharidosis type IIIB syndrome: an uncontrolled phase 1/2 clinical trial.* The Lancet Neurology, 2017. **16**(9): p. 712-720.

15. Hendriksz, C.J., et al., *Levels of glycosaminoglycans in the cerebrospinal fluid of healthy young adults, surrogate-normal children, and Hunter syndrome patients with and without cognitive impairment.* Molecular Genetics and Metabolism Reports, 2015. **5**: p. 103-106.

16. Muenzer, J., et al., *A phase I/II study of intrathecal idursulfase-IT in children with severe mucopolysaccharidosis II.* Genetics in Medicine, 2016. **18**(1): p. 73-81.

17. Shapiro, E.G., et al., *A Prospective Natural History Study of Mucopolysaccharidosis Type IIIA.* The Journal of Pediatrics, 2016. **170**: p. 278-287.e4.

18. Wijburg, F.A., et al., *Long-term safety and clinical outcomes of intrathecal heparan-N-sulfatase in patients with Sanfilippo syndrome type A.* Molecular Genetics and Metabolism, 2021. **134**(4): p. 317-322.

19. Wijburg, F.A., et al., *Intrathecal heparan-N-sulfatase in patients with Sanfilippo syndrome type A: A phase IIb randomized trial.* Molecular Genetics and Metabolism, 2019. **126**(2): p. 121-130.

20. Harmatz, P., et al., *Chemically modified recombinant human sulfamidase (SOBI003) in mucopolysaccharidosis IIIA patients: Results from an open, non-controlled, multicenter study.* Molecular Genetics and Metabolism, 2022. **136**(4): p. 249-259.

21. Jones, S.A., et al., *A phase 1/2 study of intrathecal heparan-N-sulfatase in patients with mucopolysaccharidosis IIIA.* Molecular Genetics and Metabolism, 2016. **118**(3): p. 198-205.

22. Giugliani, R., et al., *Neurocognitive and somatic stabilization in pediatric patients with severe Mucopolysaccharidosis Type I after 52 weeks of intravenous brain-penetrating insulin receptor antibody-iduronidase fusion protein (valanafusp alpha): an open label phase 1-2 trial.* Orphanet Journal of Rare Diseases, 2018. **13**(1).

23. Gentner, B., et al., *Hematopoietic Stem- and Progenitor-Cell Gene Therapy for Hurler Syndrome.* New England Journal of Medicine, 2021. **385**(21): p. 1929-1940.

24. Okur, I., et al., *Longitudinal Natural History of Pediatric Subjects Affected with Mucopolysaccharidosis IIIB.* The Journal of Pediatrics, 2022. **249**: p. 50-58.e2.

25. Harmatz, P., et al., *First-in-human in vivo genome editing via AAV-zinc-finger nucleases for mucopolysaccharidosis I/II and hemophilia B.* Molecular Therapy, 2022. **30**(12): p. 3587-3600.

26. Giugliani, R., et al., *Immune tolerance induction for laronidase treatment in mucopolysaccharidosis I.* Molecular Genetics and Metabolism Reports, 2017. **10**: p. 61-66.

27. Clarke, L.A., et al., *Long-term Efficacy and Safety of Laronidase in the Treatment of Mucopolysaccharidosis I.* Pediatrics, 2009. **123**(1): p. 229-240.

28. Giugliani, R., et al., *Disease characteristics, effectiveness, and safety of vestronidase alfa for the treatment of patients with mucopolysaccharidosis VII in a novel, longitudinal, multicenter disease monitoring program.* Orphanet Journal of Rare Diseases, 2024. **19**(1).

29. Levin, S.W., et al., *Oral cysteamine bitartrate and N-acetylcysteine for patients with infantile neuronal ceroid lipofuscinosis: a pilot study.* The Lancet Neurology, 2014. **13**(8): p. 777-787.

30. Freilinger, M., et al., *Effects of Creatine Supplementation in Rett Syndrome: A Randomized, Placebo-Controlled Trial.* Journal of Developmental & Behavioral Pediatrics, 2011. **32**(6).

31. Witters, P., et al., *D-galactose supplementation in individuals with PMM2-CDG: results of a multicenter, open label, prospective pilot clinical trial.* Orphanet Journal of Rare Diseases, 2021. **16**(1).

32. Rodan, L.H., et al., *L-Arginine Affects Aerobic Capacity and Muscle Metabolism in MELAS (Mitochondrial Encephalomyopathy, Lactic Acidosis and Stroke-Like Episodes) Syndrome.* PLOS ONE, 2015. **10**(5): p. e0127066.

33. Rodan, L.H., et al., *L-arginine effects on cerebrovascular reactivity, perfusion and neurovascular coupling in MELAS (mitochondrial encephalomyopathy with lactic acidosis and stroke-like episodes) syndrome.* PLOS ONE, 2020. **15**(9): p. e0238224.

34. Guffon, N., et al., *Long‐term safety and efficacy of velmanase alfa treatment in children under 6 years of age with alpha‐mannosidosis: A phase 2, open label, multicenter study.* Journal of Inherited Metabolic Disease, 2023. **46**(4): p. 705-719.

35. Borgwardt, L., et al., *Efficacy and safety of Velmanase alfa in the treatment of patients with alpha‐mannosidosis: results from the core and extension phase analysis of a phase III multicentre, double‐blind, randomised, placebo‐controlled trial.* Journal of Inherited Metabolic Disease, 2018. **41**(6): p. 1215-1223.

36. Borgwardt, L., et al., *Enzyme replacement therapy for alpha‐mannosidosis: 12 months follow‐up of a single centre, randomised, multiple dose study.* Journal of Inherited Metabolic Disease, 2013. **36**(6): p. 1015-1024.

37. Lund, A.M., et al., *Comprehensive long‐term efficacy and safety of recombinant human alpha‐mannosidase (velmanase alfa) treatment in patients with alpha‐mannosidosis.* Journal of Inherited Metabolic Disease, 2018. **41**(6): p. 1225-1233.

38. Borgwardt, L., et al., *Alpha-mannosidosis: correlation between phenotype, genotype and mutant MAN2B1 subcellular localisation.* Orphanet Journal of Rare Diseases, 2015. **10**(1).

39. Borgwardt, L.G., et al., *Relationship between MAN2B1 genotype/subcellular localization subgroups, antidrug antibody detection, and long‐term velmanase alfa treatment outcomes in patients with alpha‐mannosidosis.* JIMD Reports, 2023. **64**(2): p. 187-198.

40. Diaz, G.A., et al., *Long-term safety and clinical outcomes of olipudase alfa enzyme replacement therapy in pediatric patients with acid sphingomyelinase deficiency: two-year results.* Orphanet Journal of Rare Diseases, 2022. **17**(1).

41. Diaz, G.A., et al., *One-year results of a clinical trial of olipudase alfa enzyme replacement therapy in pediatric patients with acid sphingomyelinase deficiency.* Genetics in Medicine, 2021. **23**(8): p. 1543-1550.

42. Borgwardt, L., et al., *Alpha-mannosidosis: characterization of CNS pathology and correlation between CNS pathology and cognitive function.* Clinical Genetics, 2016. **89**(4): p. 489-494.

43. Naegelin, Y., et al., *Fingolimod in children with Rett syndrome: the FINGORETT study.* Orphanet Journal of Rare Diseases, 2021. **16**(1).

44. Agrawal, N., et al., *Neurofilament light chain in cerebrospinal fluid as a novel biomarker in evaluating both clinical severity and therapeutic response in Niemann-Pick disease type C1.* Genetics in Medicine, 2023. **25**(3): p. 100349.

45. Downs, J., et al., *Environmental enrichment intervention for Rett syndrome: an individually randomised stepped wedge trial.* Orphanet Journal of Rare Diseases, 2018. **13**(1).

46. Van Haren, K.P., et al., *A Phase 1 Study of Oral Vitamin D3 in Boys and Young Men With X-Linked Adrenoleukodystrophy.* Neurology Genetics, 2023. **9**(2): p. e200061.

47. De Castro-Orós, I., et al., *Assessment of plasma chitotriosidase activity, CCL18/PARC concentration and NP-C suspicion index in the diagnosis of Niemann-Pick disease type C: a prospective observational study.* Journal of Translational Medicine, 2017. **15**(1).

48. Tantawy, A.A.G., et al., *Results From a 12-Month Open-Label Phase 1/2 Study of Velaglucerase Alfa in Children and Adolescents With Type 3 Gaucher Disease.* Journal of Inborn Errors of Metabolism and Screening, 2018. **6**: p. 232640981876556.

49. Gougeon, M.-L., et al., *Cell-Mediated Immunity to NAGLU Transgene Following Intracerebral Gene Therapy in Children With Mucopolysaccharidosis Type IIIB Syndrome.* Frontiers in Immunology, 2021. **12**.
